# ODSEI Chip: An Open 3D Microfluidic Platform for Studying Tumor Spheroid‐Endothelial Interactions

**DOI:** 10.1002/advs.202410659

**Published:** 2025-01-13

**Authors:** Jooyoung Ro, Junyoung Kim, Juhee Park, Yongjun Choi, Yoon‐Kyoung Cho

**Affiliations:** ^1^ Department of Biomedical Engineering Ulsan National Institute of Science and Technology (UNIST) Ulsan 44919 South Korea; ^2^ Center for Algorithmic and Robotized Synthesis Institute for Basic Science (IBS) Ulsan 44919 South Korea

**Keywords:** co‐culture, drug‐resistant mechanism study, open microfluidics, spheroids

## Abstract

Current in vitro models of 3D tumor spheroids within the microenvironment have emerged as promising tools for understanding tumor progression and potential drug responses. However, creating spheroids with functional vasculature remains challenging in a controlled and high‐throughput manner. Herein, a novel open 3D‐microarray platform is presented for a spheroid‐endothelium interaction (ODSEI) chip, capable of arraying more than 1000 spheroids on top of the vasculature, compartmentalized for single spheroid‐level analysis of drug resistance, and allows for the extraction of specific spheroids for further analysis. As proof of concept, the crosstalk between breast cancer spheroids and vasculature is monitored, validating the roles of endothelial cells in acquired tamoxifen resistance. Cancer spheroids exhibited reduced sensitivity to tamoxifen in the presence of vasculature. Further analysis through single‐cell RNA sequencing of extracted spheroids and protein arrays elucidated gene expression profiles and cytokines associated with acquired tamoxifen resistance, particularly involving the TNF‐α pathway via NF‐κB and mTOR signaling. By targeting the highly expressed cytokines (IL‐8, TIMP1) identified, tamoxifen resistance in cancer spheroid can be effectively reversed. In summary, the ODSEI chip allows to study spheroid and endothelial interaction in various contexts, leading to improved insights into tumor biology and therapeutic strategies.

## Graphical abstract

The ODSEI chip, a novel 3D‐microarray platform, co‐cultures tumor spheroids on the vasculature, enabling drug treatment and specific spheroid extraction. The ODSEI chip demonstrates reduced tamoxifen sensitivity within spheroids when interacting with endothelial cells. Single‐cell RNA sequencing and protein arrays identify gene expression profiles and cytokines associated with tamoxifen resistance while further targeting identified cytokines effectively reduced drug resistance.

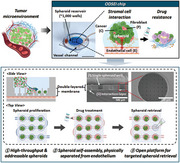

## 1 Introduction

Despite these advancements in cancer research and improved treatment outcomes, breast cancer remains the second leading cause of cancer‐related deaths among women worldwide, following lung cancer, according to 2023 statistics.^[^ ^1^ ^]^ Approximately 80% of breast cancer cases are estrogen receptor‐positive (ER+) luminal types. Estrogen stimulates the growth of ER+ breast cancer cells, leading to treatment development that either reduces estrogen levels in the body or blocks estrogen receptors on these cells to inhibit their growth. Tamoxifen, an antagonist of ERα66 that competes with estrogen for receptor binding, is one of the earliest targeted therapies for breast cancer and is also used as an adjuvant treatment.^[^ ^2^ ^]^ However, resistance to tamoxifen, as with other endocrine therapies, remains a major clinical challenge, with about 40% of initially responsive tumors experiencing recurrence, metastasis, and resistance to other chemotherapy treatments.

Tamoxifen resistance mechanisms involve the loss or deregulation of ERα and alterations in signaling pathways related to cell survival, apoptosis, and stress responses. These mechanisms may include aberrations in the ER/progesterone receptor (PgR) pathway (such as ER mutations and deregulated interactions of ER with coactivators/corepressors), overexpression of truncated ER isoforms such as ERα36 and ERα46, acquired mutations in the ligand‐binding domain of estrogen receptor 1 (ESR1), activation of alternative survival pathways (e.g., PI3K and MAPK pathways), post‐translational modifications such as histone and DNA methylation, altered transcriptional regulation, cell cycle dysregulation, and upregulated oncogenic signaling pathways.^[^ ^3^ ^]^ Furthermore, tamoxifen‐resistant tumors often exhibit distinct characteristics such as epithelial‐mesenchymal transition (EMT), metabolic reprogramming, autophagy, and acquisition of cancer stem cell (CSC) properties.^[^ ^4^ ^]^

Tumor‐activated endothelial cells (TECs) also play a significant role in chemotherapy resistance. TECs exhibit multidrug resistance (MDR) through increased expression of p‐glycoprotein and metabolic reprogramming, allowing them to maintain continuous blood perfusion and support tumor survival during anti‐cancer therapy.^[^ ^5^ ^]^ Various angiocrines, including surface adhesion proteins and cytokines such as epidermal growth factor (EGF), interleukin (IL)‐6, IL‐8, fibroblast growth factor (FGF), and Jagged‐1 secreted by TEC, can contribute to cancer resistance to anti‐cancer therapies.^[^ ^6^ ^]^ Although the role of TECs in drug resistance has been explored across various cancer types, their specific role in tamoxifen resistance in ER+ breast cancer cells has not been thoroughly investigated. Understanding these mechanisms is crucial for improving breast cancer treatment outcomes, prognoses, and therapeutic strategies. To achieve this, further research is needed to replicate the complex tumor microenvironment (TME) in preclinical models to unravel the mechanisms driving drug resistance within the TME. As demand for preclinical micro‐physiological systems to model the complex TME grows, spheroid‐on‐a‐chip technology has particularly advanced the study of 3D TMEs, enabling the creation of more in vivo‐like solid tumor structures that exhibit multicellular aggregates, distinct necrotic, quiescent, and proliferative zones, and gradients of oxygen, pH, and nutrients.^[^ ^7^ ^]^

A critical component in replicating the TME is the vascular network, which provides both direct contact and indirect influence on tumor cells through the secretion of growth factors, cytokines, nutrients, and oxygen via blood flow.^[^ ^7^ ^]^ Recent advancements in vascularized tumor‐on‐a‐chip technologies have significantly improved our ability to mimic the TME (Table S1, Supporting Information).^[^ ^8^ ^]^ These platforms employ various techniques including microfluidics, sacrificial templates, and 3D printing to study tumor progression and metastasis.^[^ ^9^ ^]^ However, many current approaches face limitations such as low to mid‐throughput capabilities, poor control over spheroid positioning, closed systems hindering spheroid extraction, and limited replication of tumor‐endothelial interactions. While some open‐system approaches have demonstrated self‐assembled spheroids with vascularization, they typically produce fewer than 10 spheroids per chip, limiting their applicability in high‐throughput studies.

The ODSEI chip addresses these limitations by offering a high‐throughput formation of over 1000 uniform‐sized, self‐assembled spheroids on a single chip. Its innovative two‐layered porous membrane design, featuring pore sizes of 10 µm and 200 µm with surface chemical modifications, allows for controlled soluble factor diffusion while maintaining physical separation between spheroids and vessels. This design better mimics the in vivo basement membrane and ECM barrier, crucial for accurately modeling initial tumor‐endothelial interactions. Furthermore, the ODSEI chip replicates tumor‐associated angiogenesis through paracrine signaling and cell‐matrix interactions,^[^ ^10^ ^]^ providing a more physiologically relevant model compared to conventional approaches that directly mix tumor and endothelial cells.

The open system architecture of the ODSEI chip facilitates easy extraction of specific spheroids for further analysis, overcoming a significant limitation of closed systems. Additionally, the compartmentalized design enables single spheroid‐level analysis, enhancing the precision of drug response studies. These features collectively extend the applicability of vascularized tumor‐on‐a‐chip systems in tumor biology research and drug discovery, offering a versatile platform for investigating complex tumor‐endothelial interactions and screening potential therapeutic agents in a more physiologically relevant context.

As proof of concept, we demonstrated the role of endothelial cells in contributing to tamoxifen resistance in tumor spheroids. Additionally, single‐cell RNA sequencing (scRNA‐seq) on spheroids retrieved from the microfluidic platform revealed gene expression profiles associated with mTOR signaling, TNF‐α via NF‐kB, and p53 pathways in tamoxifen‐resistant breast tumors. We also analyzed TME‐related cytokines and identified IL‐8 and TIMP‐1 as key cytokines highly secreted in the presence of endothelial cells. Our microfluidic platform has the potential to advance the investigation of therapeutic efficacy and metabolism within a physiologically relevant microenvironment.

## 2 Results

### 2.1 Development of the ODSEI Chip

**Figure 1:**
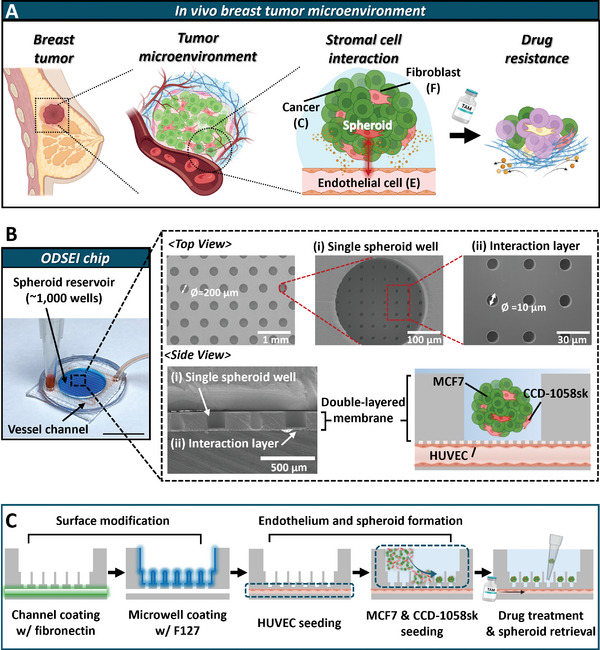
Development of the ODSEI chip for establishing the tumor spheroids and endothelium interface. A) Schematic representation of the breast tumor microenvironment (TME) in vivo, illustrating cell‐cell interactions and drug resistance at the cellular level. Drug resistance, influenced by multiple factors, leads to partial cell death (depicted in purple) within the microenvironment. B) (Left) Photograph of the ODSEI chip. The scale bar represents 1 cm. (Right) Scanning electron microscope (SEM) images of the PDMS membrane that forms part of the device. The membrane consists of two layers: i) the single spheroid wells with a diameter of 200 µm and ii) the interaction layer with a diameter of 10 µm. The scale bars are 500 µm, 1 mm, 100 µm, and 30 µm, respectively. C) Schematic workflow showing the process from spheroid culture on vasculature and retrieval from the ODSEI chip with key features: 1) Surface modification with fibronectin and F127 solution, 2) Endothelial layer is formed in the bottom channel and cancer cell and fibroblast‐mixed solution is introduced to self‐form spheroids, 3) Experiments and analysis on the ODSEI chip: spheroid proliferation, drug treatment, and spheroid retrieval.

In TME, cancer cells interact with various stromal cells, such as fibroblasts and endothelial cells, physically and chemically. These interactions contribute to progression, metastasis, and drug resistance (**Figure** **1**A). To accurately model these complex environments in vitro, it is essential to recreate the diverse cell populations in solid tumors while maintaining the reproducibility and compatibility of spheroids, which are crucial for studying tumor biology and evaluating drug efficacy.

To address this need, we developed the ODSEI chip, capable of culturing approximately 1000 self‐formed tumor spheroids composed of cancer cells and fibroblasts in a spheroid reservoir positioned on a vessel channel (Figure 1B). The chip features a two‐layered porous PDMS membrane. The upper layer contains ≈ 1000 wells, each 200 µm in diameter, called single spheroid wells, designed for spheroid cultivation. The bottom surface of these wells, known as the interaction layer, is also porous, with holes 10 µm in diameter that allow interactions between the spheroids and endothelial cells cultured on the lower microchannel layer, mimicking the endothelium (Figure 1B).

The PDMS membrane was fabricated as a single unit by injecting PDMS into the gap between glass and a PDMS replica featuring two‐story pillars, prepared using two‐step photolithography and soft lithography techniques (Figure S1, Supporting Information).^[^ ^11^ ^]^ The membrane surface of the bottom channel was pre‐coated with fibronectin (Figure S2, Supporting Information). Cancer cell and fibroblast‐mixed solution was seeded in the microwells and suspended cells were self‐assemble into spheroids, aided by prior treatment with Pluronic F127 on top of the membrane to prevent cell adhesion. This open system design allows for easy retrieval of specific spheroids, which can then be dissociated into single cells for further analysis, such as scRNA‐seq, to study interactions involved in the development of drug resistance (Figure 1C).

### 2.2 Formation and Culture of Spheroids in the ODSEI Chip

**Figure 2:**
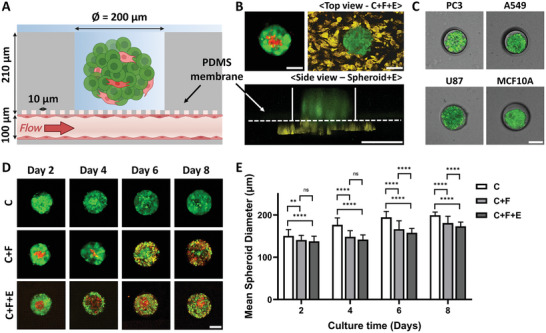
Analysis of spheroid formation and proliferation on the ODSEI platform. A) Side view of the ODSEI platform, providing detailed explanations of the device components and layout. B) Confocal images showing spheroids composed of cancer cells (C), fibroblasts (F), and endothelial cells (E) on the ODSEI device. Images are presented in both top and side views. MCF7 cells are labeled in green (CellTracker Green CMFDA), CCD‐1058sk cells in red (CellTracker Deep Red), and RFP expressing HUVEC cells in yellow for fluorescence images of the spheroids. C) Spheroid formation observed with various cell lines on the ODSEI platform. Cells were labeled with CellTracker Green CMFDA for fluorescence imaging. D,E) Proliferation of spheroids under different culture conditions, measured by confocal imaging and calculating the mean diameters of the spheroids. The conditions include cancer spheroids (C), cancer and fibroblast spheroids (C+F), and cancer and fibroblast spheroids co‐cultured with endothelial cells (C+F+E). MCF7 cells are labeled in green (CellTracker Green CMFDA) and CCD‐1058sk cells in red (CellTracker Deep Red) for fluorescence imaging. All scale bars represent 100 µm. Two‐way ANOVA was used for statistical comparison; ns., p > 0.05, ^**^p < 0.01, ^****^p < 0.0001.

The ODSEI chip's design incorporates double‐layered PDMS membrane with single spheroid wells with a diameter of 200 µm and a height of 200 µm, and an interaction layer with pores of 10 µm in diameter and 10 µm in height. This platform supports drug transport through fluid flow in the vessel channels and diffusion through the porous membrane (**Figure** **2**A). The ODSEI chip is designed to culture three cell types: MCF7 breast cancer cells (C) and CCD‐1058sk fibroblast cells (F) to make heterogeneous spheroids, and HUVEC endothelial cells (E) to make endothelium. Endothelial cells were first introduced into the underlying channel and cultured for 24 h before the mixture of MCF7 and CCD‐1058sk cells was seeded onto the membrane to form spheroids. During a 2‐h stabilization period, cancer cells and fibroblasts were trapped in the microwells and began self‐aggregating, while non‐trapped cells were washed away. Uniformly distributed spheroids on the vasculature were validated through tile‐scanned images of the entire ODSEI chip (Figure S3, Supporting Information). Z‐stack images clearly demonstrated the formation of an interface between tumor spheroids and the endothelium on day 2 (Figure 2B). Endothelial cells exhibited migration through the membrane toward the tumor spheroids while maintaining their interface during extended incubation for 7 days (Figure S4, Supporting Information). These results align with data obtained from platforms utilizing porous membranes exposed to various stimuli, such as cytokines and pro‐angiogenic factors.^[^ ^12^ ^]^

In the vessel channel, media flowed at a rate of 1.6 µL min^−1^ (0.54 dyne cm^−1^), which simulates physiologically relevant blood flow and shear stress on endothelial cells. We measured endothelial cell proliferation under these shear stress conditions and confirmed that the flow did not adversely affect cellular growth or adhesion (Figure S5, Supporting Information). Moreover, we evaluated the tight junction formation of the endothelium using immunofluorescence staining for the tight junction protein zonula occludens (ZO‐1) (Figure S6, Supporting Information). ZO‐1 expression was decreased and discontinuous in the endothelium co‐cultured with tumor spheroids, while strong and continuous ZO‐1 expression was observed in the endothelium without tumor spheroids. Additionally, the permeability of the ODSEI chip from the endothelial channel to the spheroid wells was measured using 70 kDa dextran conjugated with Texas Red dye. Consistent with decreased ZO‐1 expression in conditions with co‐culture of tumor spheroids, our ODSEI chips showed that co‐culture of tumor spheroids exhibited an average permeability of 7.5 (± 2.2) × 10⁻⁵ cm s⁻¹, which is 7.5‐fold higher than that of the mono‐culture endothelium at 1.0 (± 0.4) × 10⁻⁵ cm s⁻¹ (Figure S7, Supporting Information).

We tested four additional cell lines of different origins—prostate cancer (PC3), lung cancer (A549), brain cancer (U87), and normal breast epithelial (MCF10A) cells—to confirm the versatility of the ODSEI chip. Tumor spheroid formation was observed on Day 1 (Figure 2C), and proliferation and viability were measured over 8 days for spheroids derived from each cell line (Figure S8, Supporting Information). Our results demonstrated that the ODSEI chip efficiently formed spheroids with all tested cell types.

### 2.3 Proliferation of Spheroids in the ODSEI Chip

Spheroids were formed by seeding cells onto the membrane and incubating for 2 h to allow capture within the microwells. The membrane was coated with a 3% F127 solution to reduce cell adhesion to the PDMS, and untrapped cells were washed out before overnight incubation. The uniform‐sized wells ensured consistent spheroid distribution, with approximately 87% ± 9% of the wells occupied (Figure S9, Supporting Information). Tumor spheroid formation in the ODSEI chip was monitored at 0, 2, 6, 24, and 48 h using bright‐field microscopy (4X) and fluorescence microscopy (10X), with self‐spheroid formation becoming evident after 24 h (Figure S10, Supporting Information).

To assess the biocompatibility of the ODSEI chip, we monitored the proliferation of mono‐cultured and co‐cultured breast cancer spheroids with different cell ratios over an eight‐day period. As shown in Figure 2D,E, mono‐cultured cancer cells initially formed relatively loose spheroids, gradually becoming more compact over time. In contrast, fibroblast co‐cultured spheroids exhibited tighter aggregation from the beginning. This pattern aligns with previous studies, where fibroblasts formed a dense core within the spheroids and gradually proliferated to the surface, mingling with cancer cells.^[^ ^13^ ^]^ Previous studies have shown that spheroids can develop a core‐shell structure with stromal cells, which increases spheroid viability under harsh conditions in the TME.^[^ ^14^ ^]^

By staining cancer and fibroblast cells separately, we confirmed the formation of multicellular spheroids, where each cell type occupied distinct positions, demonstrating a specific architectural arrangement. The average diameter of the spheroids increased over time: on day 2, monocultured spheroids (C) averaged 150 µm, fibroblast co‐cultured spheroids (C+F) averaged 140 µm, and endothelial‐included co‐cultured spheroids (C+F+E) averaged 137 µm. By day 8, these sizes had grown to 199 µm, 180 µm, and 173 µm, respectively. Spheroid proliferation was further confirmed by measuring cellular metabolic activity using the MTT assay on days 2 and 8, which showed increased activity across all conditions (Figure S11, Supporting Information).

Spheroids were cultured in a mixed medium of RPMI 1640, MEM, and human endothelial medium at a 1:1:1 ratio. We verified that this mixed medium was suitable for tumor spheroid culture on the platform, as cell viability in the mixed medium was comparable to that in the original recommended medium for each cell type (Figure S12, Supporting Information). These results demonstrate the capability of the ODSEI chip to support the formation of self‐assembled spheroids with various epithelial cells and provide an interface for tumor spheroids and endothelium co‐culture under controlled conditions for over a week.

### 2.4 Increased Tamoxifen Resistance of Tumor Spheroids with Endothelium Interaction

**Figure 3:**
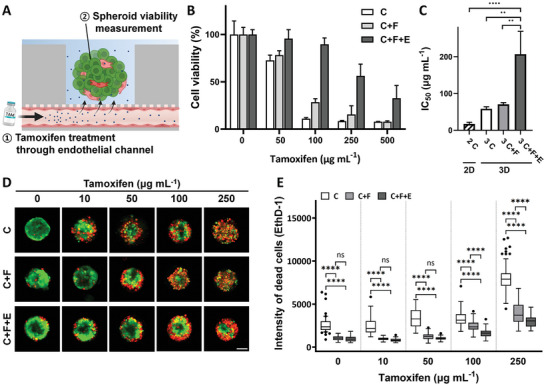
Tamoxifen toxicity experiment of tumor spheroids in the TME. A) Schematic representation of tamoxifen treatment administered through vessels in the TME. B) Cell viability of spheroids treated with tamoxifen in a dose‐dependent manner. The conditions tested include cancer spheroids (C), cancer and fibroblast spheroids (C+F), and cancer and fibroblast spheroids co‐cultured with endothelial cells (C+F+E). C) *IC*_50_ values for tamoxifen in both 2D monolayer cultures and 3D spheroids under different conditions, measured using the MTT assay. The *IC*_50_ values are as follows: 19.63 µg mL^−1^ for 2D monolayer culture, 57.65 µg mL^−1^ for C spheroids, 70.24 µg mL^−1^ for C+F spheroids, and 213.3 µg mL^−1^ for C+F+E spheroids. D) Live/dead assay with Calcein‐AM for live cells (represented with green) and EthD‐1 for dead cells (represented with red) in spheroids from different drug treatment conditions. E) The intensity of the dead cells, stained with EthD‐1 were compared in each drug concentration and culture condition. All scale bar sizes are 100 µm. One‐way and two‐way ANOVA for statistical comparison; ns., p > 0.05; ^*^p < 0.05, ^**^p < 0.01, ^***^p < 0.001, ^****^p < 0.0001.

To evaluate drug efficacy using the ODSEI chip, we conducted studies with tamoxifen, a commonly used chemotherapy drug for estrogen receptor‐positive breast cancer, as a proof of concept. We investigated the impact of endothelial cells on tamoxifen resistance in cancer spheroids (**Figure** **3**A).

We compared the tamoxifen reactivity in spheroids cultured under three different conditions: monoculture (C), fibroblast co‐culture (C+F), and endothelial‐included co‐culture (C+F+E). The dose‐dependent response of these spheroids after 24 h of tamoxifen treatment is shown in Figure 3B. Additionally, we analyzed the *IC*_50_ values across different culture conditions, including monolayer cancer cell cultures. The *IC*_50_ values were determined by non‐linear regression fitting of the log [tamoxifen] versus cell viability curve using absorbance measured through the MTT assay in GraphPad Prism (Figure S13, Supporting Information).

Following tamoxifen treatment, the *IC*_50_ values were 17.36 µg mL^−1^ for monolayer cancer cells, 123.6 µg mL^−1^ for monoculture (C), 147.9 µg mL^−1^ for fibroblast co‐culture (C+F), and 213.3 µg mL^−1^ for endothelial‐included co‐culture (C+F+E) spheroids (Figure 3C). As expected, the 3D cell culture models exhibited increased drug resistance compared to 2D models, likely due to the reduced penetration of drugs into the core of 3D structures, aligning with previous reports.^[^ ^15^ ^]^ Notably, the highest *IC*_50_ value was observed in the C+F+E spheroids, approximately 1.44 times higher than in C+F spheroids, indicating enhanced tamoxifen resistance due to endothelial cell interaction. Additionally, we have carried out longer tamoxifen treatment to the C+F+E spheroids at *IC*_50_ value of 213.3 µg mL^−1^ for 24, 48, and 72 h. Compared to the 24 h condition, culturing spheroids for over 48 and 72 h showed a slight decrease in viability, as measured with MTT assay and imaged with Calcein‐AM and EthD‐1 (Figure S14, Supporting Information).

We also performed live/dead imaging to assess individual spheroid viability following tamoxifen treatment (Figure 3D,E). Using the dead cell marker EthD‐1, we quantified the ratio of dead cells in each spheroid based on the dye intensity. The single‐spheroid viability results were consistent with those obtained from the bulk MTT assay (Figure 3B). Overall, our dose‐dependent tamoxifen sensitivity screening of both bulk and individual tumor spheroids demonstrated the role of endothelial cells in increasing tamoxifen resistance in tumor spheroids.

### 2.5 Single‐Cell RNA Sequencing of Tumor Spheroids Retrieved from ODSEI Chip

**Figure 4:**
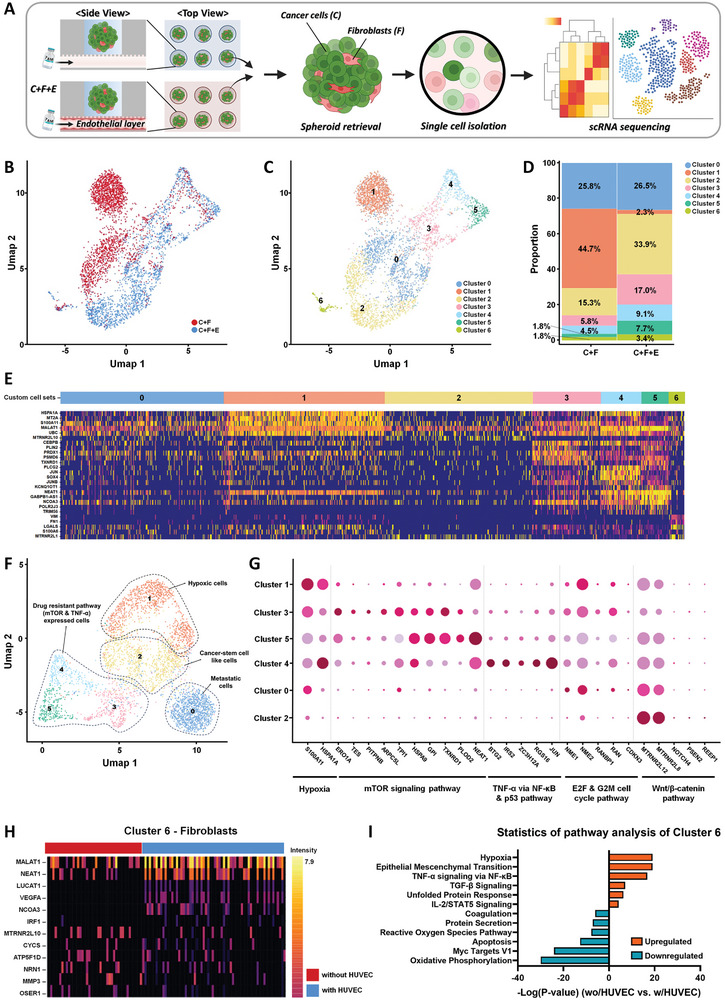
Transcriptomic cell atlas of tumor spheroids with and without endothelium interaction following tamoxifen exposure. A) Schematic workflow showing the process from spheroid culture and retrieval from the ODSEI chip to single‐cell RNA sequencing. B) Uniform Manifold Approximation and Projection (UMAP) plot of all cells (n = 829) that passed quality control, colored by their sample of origin. Samples include cancer and fibroblast spheroids (C+F) and cancer and fibroblast spheroids co‐cultured with endothelial cells (C+F+E). C) UMAP plot showing the distribution of breast cancer cells and fibroblasts, colored by specific clusters. D) Proportion of each cluster in the different samples, indicating the distribution of cell types across conditions. E) Heatmap displaying cluster‐specific gene expression, with yellow indicating high expression and purple indicating low expression. F) UMAP plot highlighting subclusters of breast cancer cells. Highly expressed pathways enriched from the differentially expressed genes of each breast cancer subcluster to all other subclusters. G) Dot plot illustrating the relative expression of selected marker genes associated with enriched pathways in different breast cancer subtypes. The color intensity scale represents the average expression level per cell, while the size scale indicates the percentage of cells expressing each gene within the specific cell type. H) Heatmap showing distinct gene signatures of fibroblasts under different culture conditions, focusing on culture condition‐specific gene expressions. I) Pathway analysis comparing fibroblasts co‐cultured with endothelial cells to those cultured without endothelial cells, highlighting differentially regulated pathways.

To explore how endothelial cell interaction affects tamoxifen resistance in tumor spheroids, we performed scRNA‐seq on tumor spheroids cultured without (C+F) and with (C+F+E) endothelial cells following treatment with tamoxifen at 213 µg mL^−1^, which corresponds to the *IC*_50_ value for spheroids co‐cultured with endothelial cells after 72 h. Tumor spheroids treated with tamoxifen were retrieved from the ODSEI chip and dissociated into single cells to analyze the cellular diversity underlying chemoresistance. Transcriptomes from 4167 cells were initially sequenced, with 829 cells (335 from spheroids without endothelium and 494 from spheroids with endothelium) passing quality control for subsequent analysis (**Figure** **4**A; Figure S15, Supporting Information).

Using principal component analysis (PCA) of transcriptional variations from highly variable genes, scRNA‐seq data revealed seven distinct cell clusters (Figure 4B). Differentially expressed genes (DEGs) were identified between each pair of clusters to characterize these groups. Unsupervised clustering and uniform manifold approximation and projection (UMAP) were employed to visualize cellular composition and proportions based on cell type and sample origin. Established markers for breast cancer cells (*BRCA1, MAP3K1, BMPR1A*) and fibroblasts (*FN1, VIM, COL6A2*) were used to classify the clusters (Figure S16, Supporting Information). Breast cancer cells were predominantly found in clusters 0–5, while fibroblasts were primarily in cluster 6 (Figure 4B,C). Notably, the proportion of each cluster varied by sample, but overall, breast cancer cells were more abundant than fibroblasts (Figure 4D).

The expression patterns of marker genes were visualized through a heatmap (Figure 4E). Most breast cancer cells (clusters 0–5) exhibited high levels of hypoxia‐related genes, including *HSPA1A, MT2A, S100A11, MALAT1*, and *UBC*. Clusters 3 and 5 showed elevated expression of genes associated with cancer development and progression, such as *CEBPB, PLIN2, PRDX1, PSMD6*, and *TXNRD1*. Cluster 4 was characterized by upregulated tumorigenesis‐related genes, including *PLCG2, JUN, SOX4*, and *JUNB*. Cluster 5 had high expression of genes involved in drug resistance regulation, such as *GABPB1‐AS1, NCOA3, POLR2J3*, and *TRIM56*. Fibroblast markers like *VIM, FN1*, and *LGALS* were prominent in cluster 6. Furthermore, detailed expression patterns within each breast cancer subcluster were identified.

### 2.6 ODSEI‐Derived Single Cells Expressed Paracrine Signaling Pathways Through sc‐RNA Seq After Tamoxifen Exposure

We identified distinct subclusters of breast cancer cells with specific expression patterns: metastatic breast cancer (cluster 0), hypoxic signature upregulated breast cancer (cluster 1), cancer stem‐cell‐like breast cancer (cluster 2), breast cancers expressing mTOR signaling pathways (clusters 3 and 5), and breast cancers expressing TNF‐α via NF‐kB and p53 pathways (cluster 4) (Figure 4F).

Comparative analysis of cluster proportions under different conditions revealed varying breast cancer characteristics. Cluster 0, associated with metastatic breast cancer, showed similar proportions of 25.8% without endothelium and 26.5% with endothelium. Cluster 1, which exhibited a hypoxic signature, displayed a significantly higher proportion (44.7%) without endothelial cells compared to 2.3% with endothelial cells. In the case of cluster 2, which represents cancer stem‐cell‐like breast cancer, tumors with endothelial cells exhibited a higher proportion (33.9%), nearly double that of tumors without endothelium. Clusters 3 and 5, characterized by mTOR signaling pathway expression, had a significantly higher proportion of tumors with endothelial cells (22.8%) than those without endothelial cells (9.5%). Cluster 4, associated with TNF‐α via NF‐kB and p53 pathway expression, showed proportions of 4.5% without endothelium and 9.1% with endothelium (Figure 4D).

Given that breast cancer subpopulations show distinct functional markers, we identified DEGs and enriched pathways to further characterize these subclusters (Figure 4G). Clusters 3 and 5 exhibited significant expression of mTOR signaling‐related genes, including *S100A11, HSPA1A, ERO1A, TES, PITPNB, ARPC5L, TPI1, HSPA9, GPI, TXNRD1, PLOD2*, and *NEAT1*. The mTOR (mammalian target of rapamycin) signaling pathway, a crucial cellular network regulating various processes, showed heightened activity in our co‐culture conditions. In cancer environments, hyperactivation of mTOR signaling is common, promoting tumor growth, mutations, and poor survival outcomes in breast cancer. Our data indicate that when mTOR signaling is activated in cancer cells within the TME, endothelial proliferation activity increases, driven by the upregulation of pro‐angiogenic factors such as vascular endothelial growth factor (VEGF) and IL‐8.^[^ ^16^ ^]^

Cluster 4 showed high expression of genes involved in the TNF‐α via NF‐kB and p53 pathways, such as *BTG2, IRS2, ZC3H12A, RGS16*, and *JUN*. The interplay between TNF‐α via NF‐kB and p53 emerged as a significant factor in developing chemoresistance and resistance to endocrine therapy. TNF‐α‐mediated activation of NF‐kB led to increased expression of various angiogenic factors, including VEGF, IL‐8, and basic fibroblast growth factor (bFGF), promoting tumor angiogenesis. This NF‐kB activation typically promotes drug resistance and cell survival.^[^ ^16^, ^17^ ^]^ Interestingly, while p53 activity is generally associated with promoting apoptosis and sensitivity to chemotherapy, our findings align with previous studies showing that TNF‐α mediated activation of NF‐kB can stimulate p53 gene transcription in MCF7 cells, leading to a transcriptionally inactive form of p53.^[^ ^18^ ^]^

Additionally, we identified genes associated with cell proliferation pathways, specifically E2F and G2 M cell cycles, including *NME1, NME2, RANBP1, RAN*, and *CDKN3*, as well as the Wnt/β‐catenin pathway, involving *MTRNR2L12, MTRNR2L8, NOTCH4, PSEN2*, and *REEP1*. The Wnt/β‐catenin pathway, involved in tumor initiation and progression, cancer stem cell maintenance, and drug resistance,^[^ ^19^ ^]^ also showed altered activity in our co‐culture conditions. This pathway's modulation in the presence of endothelial cells suggests a potential mechanism for enhanced drug resistance in the tumor spheroids. Therefore, the high expression of mTOR, TNF‐α via NF‐kB, and Wnt/β‐catenin pathways in clusters 3, 4, and 5 suggest a link to drug resistance through the formation of an autocrine inflammatory loop.

To understand the transcriptional profile of fibroblasts cultured in tumor spheroids under different conditions, we conducted a DEG analysis of fibroblasts with or without endothelial interaction (Figure 4H). In fibroblasts cocultured with endothelial cells, we observed significant upregulation of genes associated with cancer progression (*MALAT1, NEAT1*), vascular endothelial growth factor (*VEGFA*), and proinflammatory cytokines (*NCOA3, IRF1*). Conversely, fibroblasts without endothelial interaction showed upregulation of genes related to receptor antagonist activity (*MTRNR2L10*), apoptosis initiation (*CYCS*), oxidative phosphorylation (*ATP5F1D*), cellular response to hydrogen peroxide (*OSER1*), and extracellular matrix breakdown (*MMP3*).

Pathway enrichment analysis revealed that fibroblasts cocultured with endothelial cells significantly upregulated TNF‐α via NF‐kB, TGF‐β, and IL‐2/STAT5 signaling pathways compared to those without endothelial interaction. We observed that TNF‐α induced an inflammatory phenotype through NF‐kB activation, leading to increased production of pro‐tumorigenic chemokines such as CCL2, CXCL8, and CCL5.^[^ ^20^ ^]^ Additionally, the TGF‐β signaling pathway in fibroblasts played a central role in fibroblast activation, metabolic reprogramming, and tumor‐promoting functions, contributing to the remodeling of the TME. We also noted that IL‐2 signaling in fibroblasts induced autophagy, promoting the proliferation and survival of these cells. The activation of STAT5A and STAT5B, key transcription factors downstream of IL‐2 receptor signaling, further underscores the complex interplay between different cell types in our model. In contrast, pathways related to coagulation, reactive oxygen species, and apoptosis were downregulated in fibroblasts with endothelial cells (Figure 4I).

These findings collectively demonstrate that the presence of endothelial cells in our ODSEI chip model significantly alters the gene expression landscape of tumor spheroids, activating multiple signaling pathways that contribute to drug resistance. This deeper understanding of the molecular mechanisms underlying endothelial‐tumor interactions provides valuable insights for developing more effective therapeutic strategies against drug‐resistant breast cancer.

### 2.7 Cytokine Secretion of Tumor Spheroids with Endothelium Interaction in Tamoxifen Response

**Figure 5:**
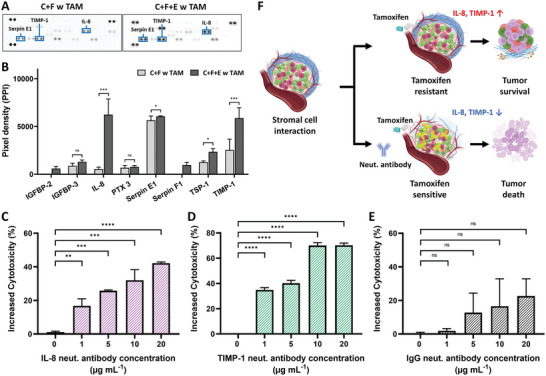
Cytokine array and neutralization experiments to elucidate the role of endothelium interaction in tamoxifen resistance of tumor spheroids. A,B) Cytokine arrays showing the secretions from tumor spheroids treated with tamoxifen (C+F with tamoxifen) and tumor spheroids co‐cultured with endothelium (C+F+E with tamoxifen) following treatment with 213.3 µg mL^−1^ of tamoxifen. C–E) Comparison of tamoxifen cytotoxicity after neutralization of specific cytokines using neutralizing antibodies on the ODSEI chip: IL‐8 (C), TIMP‐1 (D), and IgG as a control (E). F) Schematic illustration of the mechanism of tamoxifen resistance in ER+ tumors facilitated by endothelium interaction, as revealed using the ODSEI chip. One‐way and two‐way ANOVA were used for statistical comparisons; ns., p > .05, ^*^p < .05, ^**^p < .01, ^***^p < .001.

Stromal cells, such as fibroblasts and endothelial cells, play a crucial role in developing chemoresistance by secreting soluble factors in a paracrine manner.^[^ ^21^ ^]^ To investigate the cytokines involved in this process, particularly those derived from endothelial cells contributing to tamoxifen resistance, we conducted a human cytokine antibody array. The array analyzed cytokine expression in co‐cultured spheroids composed of tumor cells and fibroblasts and spheroids with added endothelial cells following tamoxifen exposure. After normalizing the data against a reference blot for each membrane, we analyzed the pixel intensity for each cytokine (**Figure** **5**A,B). Cytokines such as EGF and VEGF were present in the mixed culture medium and were excluded from the final analysis (Figure S17, Supporting Information).

The cytokine array revealed that tumor spheroids with endothelial interaction exhibited higher levels of inflammatory proteins, such as IL‐8 and Pentraxin‐3 (PTX3), pro‐angiogenic proteins including insulin‐like growth factor binding proteins (IGFBP‐2 and IGFBP‐3), and anti‐angiogenic proteins like serine protease inhibitors (Serpin E1 and F1), tissue inhibitor of metalloproteinase‐1 (TIMP‐1), and thrombospondin‐1 (TSP‐1). Notably, IL‐8 and TIMP‐1 showed significantly higher expression levels in spheroids with endothelial cells than those without.

IL‐8, a chemokine produced by macrophages, epithelial, and endothelial cells, is known for its role in cancer progression and metastasis. It is commonly overexpressed in the breast cancer microenvironment, promoting tumor growth and enhancing invasive and metastatic properties in both hormone‐positive and hormone‐negative patients.^[^ ^22^ ^]^ However, the role of increased IL‐8 secretion in the TME due to endothelial cells and its correlation with tamoxifen resistance in breast cancer has not been previously elucidated. TIMP‐1, on the other hand, inhibits matrix metalloproteinases (MMPs) involved in extracellular matrix (ECM) remodeling. In breast cancer, TIMP‐1 acts as a growth factor, promoting cell proliferation and tumorigenesis, inhibiting apoptosis, and regulating ECM degradation, which can affect drug penetration.^[^ ^23^ ^]^

Given the prominent roles of IL‐8 and TIMP‐1 in the interaction between endothelium and tumor spheroids, we hypothesized that these cytokines are key contributors to tamoxifen resistance in the TME. To confirm this, we verified that neutralizing antibodies alone do not affect cell toxicity in the absence of tamoxifen treatment (Figure S18, Supporting Information). IL‐8 and TIMP‐1 were neutralized using specific antibodies at various concentrations of 0, 1, 5, 10, 20 µg mL^−1^ in the mixed culture medium and incubated the spheroids for 48 h. Following a 24‐h exposure to tamoxifen at a concentration of 213 µg mL^−1^, we observed that tamoxifen cytotoxicity increased by 42.18% with IL‐8 neutralization and by 70.20% with TIMP‐1 neutralization (Figure 5C,D).

We also investigated the role of Serpin E1, another cytokine highly expressed in the array, by using a Serpin E1 neutralizing antibody. Serpin E1 is involved in signal transduction, cell adhesion, and migration. After treatment with Serpin E1 neutralizing antibody, tamoxifen cytotoxicity increased by 9.59%, 51.18%, 59.51%, and 58.64% as the neutralizing antibody concentration increased from 1 to 20 µg mL^−1^ (Figure S19, Supporting Information). To confirm non‐specific binding effects, we used an IgG neutralizing antibody at similar range of concentrations (0–20 µg mL^−1^), which resulted in a lower increase in tamoxifen cytotoxicity (1.92%, 12.70%, 16.55%, 22.63%) (Figure 5E).

The significant increase in tamoxifen cytotoxicity upon neutralizing IL‐8, TIMP‐1, and Serpin E1 suggests that these specific proteins mediate tamoxifen resistance. These findings collectively indicate that endothelial cells secrete a variety of cytokines, including IL‐8, TIMP‐1, and Serpin E1, into the TME, thereby contributing to the acquired resistance of tumor spheroids to tamoxifen. This study enhances our understanding of the role of endothelial cells in tamoxifen resistance in breast cancer through paracrine signaling mechanisms (Figure 5F).

## 3 Discussion

One of the major challenges in cancer treatment is the development of chemoresistance, which often leads to more aggressive tumors with an increased ability to metastasize.^[^ ^24^ ^]^ Tumors exist in close association with their surrounding microenvironment, and the dynamics of this heterogeneous ecosystem significantly influence the sensitivity of tumor cells to drug treatments. Cancer chemoresistance is typically attributed to the heterogeneity within the cancer cell population. A crucial factor contributing to this resistance is the diversity within the TME, including the presence of stromal cells, varying oxygen levels, and the acidity of the environment.^[^ ^25^ ^]^

Our study introduces an innovative open microfluidic system, the ODSEI chip, designed to create an interface between tumor spheroids and endothelium in a 3D environment. The ODSEI chip facilitates high‐throughput spheroid formation under controlled conditions, eliminating the need to incorporate 3D tumor spheroids from external platforms, and allows for the easy retrieval of specific spheroids (Figure S20, Supporting Information). In our work, we successfully generated approximately 1000 tumor spheroids using the ODSEI chip and utilized these spheroids to explore the mechanisms of chemotherapy resistance in interactions with endothelial cells. Through scRNA‐seq of retrieved spheroids, we were able to investigate the molecular effects of endothelial cells on tumor spheroids and identify enriched cellular pathways. Additionally, we analyzed the secreted proteins from the cultured medium within the device to pinpoint highly expressed soluble factors that serve as key mediators of cellular signaling in the TME.

A key innovation of the ODSEI chip lies in its dual‐layer PDMS membrane, which presents distinct advantages for facilitating co‐culture and interaction between endothelial cells and tumor spheroids. The membrane's design, featuring varying pore sizes of 10 µm and 200 µm, along with surface chemical modifications, enables precise control over cellular interactions. The smaller pores facilitate chemical signaling between layers, while the larger pores support physical interactions and nutrient exchange, essential for maintaining viable co‐cultures. This configuration allows for high‐throughput arraying of over 1000 tumor spheroids atop a vascular network, significantly enhancing experimental efficiency and statistical robustness. Furthermore, the compartmentalized design permits single spheroid‐level analysis, providing detailed insights into individual spheroid responses. The open‐system architecture facilitates easy extraction of specific spheroids for further analysis, overcoming limitations inherent in closed systems. The versatile fabrication method, based on advanced lithography techniques, allows precise control over pore sizes,^[^ ^26^ ^]^ enabling tailored exploration of both chemical and physical interactions within co‐cultures. By more accurately mimicking the in vivo TME—where the basement membrane and ECM initially separate endothelial cells from tumors—this design offers a physiologically relevant platform for studying tumor angiogenesis and metastasis. Collectively, these features position the ODSEI chip as a powerful tool for advancing our understanding of tumor biology and developing novel therapeutic strategies.

Our findings align with previously reported mechanisms of breast cancer resistance observed in vivo, where cytokines produced by breast cancer cells following chemotherapy activate the Wnt/β‐catenin and NF‐kB pathways. This activation promotes the production and secretion of cytokines by breast cancer cells, creating an autocrine inflammatory loop that supports the growth of chemoresistant cells.^[^ ^19^ ^]^ IL‐8 is known to be associated with EMT and tumor progression within the TME. When chemotherapy targets cancer cells, it often triggers a stromal reaction, leading to TNF‐α production by stromal and endothelial cells.^[^ ^27^ ^]^ By blocking IL‐8 in the TME, we can disrupt this autocrine positive feedback loop between IL‐8 and tumor cells, thereby enhancing drug sensitivity. In this study, we specifically targeted IL‐8, the most highly expressed protein in tamoxifen‐treated HUVECs co‐cultured conditions, alongside TIMP‐1 and Serpin E1, which encode proteins involved in ECM remodeling by inhibiting MMP activity. Targeting these soluble factors proved effective in increasing tumor sensitivity to anticancer therapy, as the paracrine survival network upregulated in endothelial co‐cultured conditions provides advantages to cancer cells that activate chemoprotective programs in response to tamoxifen.

Furthermore, we observed distinct gene expression profiles in fibroblasts within tumor spheroids that interacted with endothelial cells. These fibroblasts showed upregulation of *MALAT1, NEAT1, LUCAT1, VEGFA, NCOA3*, and *IRF1*, which are associated with pathways such as EMT, TNF‐α via NF‐kB signaling, TGF‐β signaling, and IL‐2/STAT5 signaling. These changes in fibroblasts may contribute synergistically to inducing chemoresistance in tumors. The proposed mechanism of tamoxifen resistance in cancer‐associated fibroblasts involves secreted soluble factors and secretory factor‐mediated drug resistance.^[^ ^28^ ^]^ Chemokines and cytokine‐signaling pathways drive fibroblasts to remodel the ECM within the TME, creating a supportive environment for the transformation of tumor cells into EMT‐like phenotypes. This process also involves the production of growth factors, exosomes, desmoplastic reactions, and cytokines, which protect cancer cells from drug‐induced apoptosis.^[^ ^19^, ^22^ ^]^ The secretion of paracrine regulatory factors such as TGF‐β, TNF‐α, and IL‐2 by fibroblasts promotes the development of a microenvironment conducive to tumor angiogenesis, metastasis, and therapeutic resistance, ultimately supporting tumor growth.

## 4 Conclusion

The ODSEI chip effectively establishes an in vitro model of the ER+ breast cancer TME to elucidate the role of endothelium in acquired tamoxifen resistance. By co‐culturing tumor spheroids with endothelial cells, the ODSEI chip allows for the observation of how secreted cytokines from endothelial cells influence fibroblasts and breast cancer cells. This interaction promotes the secretion of paracrine factors from both fibroblasts and cancer cells, which subsequently activate drug resistance‐related pathways in breast cancer cells, thereby diminishing the efficacy of tamoxifen. Our results using the ODSEI chip suggest potential targets for novel treatments for drug‐resistant breast cancer patients.

The ODSEI chip represents a versatile platform with significant potential for expanding cancer research beyond breast cancer models. The adaptable design enables precise control over spheroid characteristics, including size, shape,^[^ ^29^ ^]^ and positioning, which facilitates the simultaneous cultivation of diverse tumor spheroids and organoids. This flexibility supports the development of organotypic cancer models^[^ ^9^, ^30^ ^]^ and investigation of organotrophic metastasis.^[^ ^9a,f,g,m^ ^]^ Future optimization strategies for the platform include several promising approaches. First, integrating additional cell types such as immune cells or stromal components could more comprehensively recapitulate the TME's complexity. Second, implementing dynamic flow systems with controllable rates would better simulate in vivo physiological conditions and enable sophisticated pharmacokinetic studies. Third, incorporating real‐time monitoring capabilities through advanced biosensors or imaging technologies could provide non‐invasive, continuous cellular response tracking. Lastly, developing automated handling systems would enhance throughput, making large‐scale drug screening more efficient and accessible.

In the context of personalized medicine, the ODSEI chip offers unprecedented opportunities for patient‐specific cancer research. By incorporating patient‐derived tumor organoids generated from biopsy or surgical samples,^[^ ^31^ ^]^ along with autologous immune cells,^[^ ^32^ ^]^ and patient‐specific decellularized extracellular matrix,^[^ ^33^ ^]^ researchers can create highly personalized TME models. This approach enables real‐time monitoring of anticancer drug responses in vitro, potentially improving the correlation between experimental and clinical drug efficacy. Such a system could provide comprehensive evaluations of personalized therapeutic strategies and facilitate the identification of disease‐specific biomarkers to optimize clinical outcomes.^[^ ^34^ ^]^

These advancements position the ODSEI chip as a transformative technology in cancer research, offering unprecedented insights into tumor biology, drug response mechanisms, and personalized therapeutic strategies. By bridging the gap between traditional in vitro models and complex in vivo systems, this platform has the potential to accelerate drug discovery and precision medicine approaches.

## 5 Experimental Section

Experimental procedures and methodological specifics are delineated in the accompanying Supporting Information.

## Conflict of Interest

The authors declare no conflict of interest.

## Supporting information



Supporting Information

## Data Availability

The data that support the findings of this study are available from the corresponding author upon reasonable request.
