# Supplementary material for: ODSEI Chip: An Open 3D Microfluidic Platform for Studying Tumor Spheroid‐Endothelial Interactions
Source: Adv Sci (Weinh). 2025 Jan 13;12(13):2410659. doi: 10.1002/advs.202410659 (PMC11967799; doi:10.1002/advs.202410659)
Supplement: Supplementary file 1 — Supporting Information [file ADVS-12-2410659-s001.docx]

Supporting Information

**ODSEI Chip: An Open 3D Microfluidic Platform for Studying Tumor Spheroid-Endothelial Interactions**

*Jooyoung Ro*, *Junyoung Kim*, *Juhee Park*, *Yongjun Choi* *and Yoon-Kyoung Cho**

J. Ro, J. Kim, Y-K. Cho

Department of Biomedical Engineering,
Ulsan National Institute of Science and Technology (UNIST),
Ulsan, 44919, Korea

Email: ykcho@unist.ac.kr

J. Ro, J. Kim, J. Park, Y. Choi, Y-K. Cho

Center for Algorithmic and Robotized Synthesis,
Institute for Basic Science (IBS),
Ulsan, 44919, Korea

Email: ykcho@unist.ac.kr

**Experimental Section**

*Microfluidic chip design and fabrication*: The microfluidic device featured a double-layered PDMS membrane with two porous layers, 10 μm and 200 μm thickness, respectively. PDMS blocks with microchannels were 600 μm in width and 70 μm in height. 25 mm outer and 14 mm inner doughnut-shaped PDMS (5:1) blocks were used to create the reservoir on top of the membrane and channel. The microchannel PDMS block, PDMS membrane, and PDMS reservoir were bonded together using O_2_ plasma treatment (80 W plasma power, 80 sccm O_2_) for 1.5 minutes. The inlets and outlets connected to the channel were made with 2 mm punches. The assembled device was then sterilized with 99.9% ethyl alcohol (0009-05027, Samchun Chemicals) and exposed to ultraviolet irradiation for 1 hour before solution treatment and cell seeding. The detailed fabrication processes of microfluidic chip components are explained in **Figure S1**, Supporting Information.

*Cell culture:* All cells were cultured at 37℃ in the presence of 5% CO_2_. Breast cancer MCF7 cells (HTB-22, ATCC) were maintained in Roswell Park Memorial Institute (RPMI)-1640 medium (11875119, Gibco) supplemented with 10% (v v^-1^) fetal bovine serum (FBS) and 1X antibiotic-antimycotic solution (15240062, Gibco). Normal breast fibroblast CCD-1058sk cells (CRL-2071, ATCC) were cultured in minimum essential medium (MEM) (11095080, Gibco), also supplemented with 10% (v v^-1^) FBS and 1X antibiotic-antimycotic solution. Human umbilical vein endothelial cells (HUVEC) (C0035C, Thermo Fisher, Waltham, MA, USA) and RFP expressing HUVECs (cAP-0001RFP, Angio-proteomie) were cultured in complete human endothelial cell medium (H1168, Cell Biologics) supplemented with growth factors including VEGF, heparin, EGF, FGF, hydrocortisone, L-glutamine, antibiotic-antimycotic solution, and FBS.

Glioblastoma U-87 MG cells and lung cancer A549 cells were cultured in Dulbecco’s modified Eagle’s medium (DMEM) (11965092, Gibco) supplemented with 10% (v v^-1^) FBS and 1X antibiotic-antimycotic solution. Prostate cancer PC3 cells were maintained in RPMI-1640 medium supplemented with 10% (v v^-1^) FBS and 1X antibiotic-antimycotic solution. Normal breast epithelial MCF10A cells were cultured in DMEM/F12 Ham’s mixture supplemented with 10% (v v^-1^) FBS and 1X antibiotic-antimycotic solution.

*Vasculature and spheroid formation in the ODSEI chip*: To replicate 3D tumor structures, spheroids were formed within the 200 μm diameter wells of the PDMS membranes. After fibronectin coating of the endothelial channels (**Figure S2,** Supporting Information), the PDMS membranes were coated with a 3% (w w^-1^) solution of Pluronic F127 (P2443, Sigma) at room temperature for 4 hours to prevent cell adhesion to the membrane surface. Subsequently, the coating solution was thoroughly washed from the device using PBS (10010023, Gibco). After coating the surfaces of the device, endothelial cells were seeded and cultured for 24 hours. A mixture of MCF7 cells and CCD-1058sk cells, at a 3:1 ratio, was then introduced into the membrane area of the chip. The seeding densities for MCF7 and CCD-1058sk cells were 9 × 10⁵ and 3 × 10⁵ cells per chip, respectively. The cells were allowed to stabilize for 2 hours, followed by a gentle wash with culture medium to remove any cells not captured within the wells on the membrane. During co-culture, endothelial cells, breast cancer cells, and fibroblasts were maintained in a mixed culture medium composed of an equal ratio (1:1:1) of their respective media. After seeding, all cells were cultured at 37°C in a 5% CO₂ environment. The culture medium in the reservoir was refreshed through the perfusion underlying channel every 24 hours using a gravity-driven directional flow.

To verify the uniform distribution of tumor spheroids within the ODSEI chip, bright-field images from three chips (average of 576 wells per chip) were analyzed to measure the spheroid coverage in individual wells. For further experiments involving specific spheroids, tumor spheroids were isolated using a Kuiqpick micromanipulator (NeuroInDx) equipped with a 200 µm capillary. Optimized vacuum settings enabled the precise extraction of single spheroids from the membrane wells into a small volume of medium. The extracted spheroids were then transferred to individual tubes and stored in culture medium.^[1]^

*MTT Assay*: Cell proliferation and viability were assessed using the MTT assay (V13154, Invitrogen). For monolayer cell proliferation measurements, each cell type was seeded in a 24-well plate at a density of 3 ⅹ 10^4^ cells per well. For spheroid proliferation and viability measurements, cells were seeded at a total density of 1.2 ⅹ 10^6^ cells per chip. The cells were cultured for 24 hours in either a mixed culture medium or the manufacturer-recommended medium at 37℃ with 5% CO_2_. Fresh medium (200 μL) and MTT stock solution (20 μL) were added to each well. After 4 hours of incubation at 37℃ with 5% CO_2_, 200 μL of SDS-HCl solution was added to each well and incubated for an additional 4 hours at 37℃. Absorbance was measured at 570 nm using a TECAN plate reader (M2000 PRO, Tecan, Männedorf, Switzerland).

*Live/dead assay:* The viability of cells forming multicellular spheroids was evaluated using the live/dead viability/cytotoxicity kit (L3224, Invitrogen). Ethidium homodimer-1 (EthD-1), which enters cells with compromised membranes and binds to DNA, emits red fluorescence, indicating dead cells. Calcein AM interacts with intracellular esterases, emitting green fluorescence to indicate live cells. EthD-1 (1 μM) and Calcein AM (0.5 μM) were mixed with cell culture media, and cells were incubated with this reagent mixture for 30 minutes at 37℃ with 5% CO2. Labeled cells were then identified and imaged using confocal microscopy (TCS SP8, Leica Microsystems) with excitation/emission wavelengths of 494/517 nm for Calcein AM and 528/617 nm for EthD-1.

*Cell labeling for fluorescence imaging:* For confocal imaging, MCF7 cells were labeled with CellTracker Green CMFDA dye (1 μM) (C2925, Thermo Fisher) and CCD-1058sk cells were tracked using CellTracker Deep Red (1 μM) (C34565, Thermo Fisher). The labeled cells were visualized using a confocal microscope (confocal microscopy (TCS SP8, Leica Microsystems), with excitation/emission wavelengths set at 492/517 nm for CMFDA and 630/650 nm for Deep Red.

*Single-cell RNA-sequencing:* For the scRNA-seq analysis, spheroids were collected from a total of 10 chips. 5 experimental replicates for each experimental condition (spheroids grown with HUVECs and without HUVECs), and dissociated into single cells with gentle pipetting. The cell count of the spheroids on the chips after washing and culture of 72 hours was approximately 3 × 10^5^ cells per chip, due to the cell loss during the processes. Subsequently, scRNA-seq was performed by Ebiogen (Korea) to compare the gene expression profiles of C+F and C+F+E spheroids, and the libraries were prepared using the 10X Genomics technology. The Cell Ranger Single-Cell Software Suite (V6.1.2) was used for sample demultiplexing, barcode processing, and single-cell 3’ gene counting. The WinSeurat program provided by E-biogen and ‘Cellenics,’ an open-source cloud-based platform for scRNA-seq data, was employed for further data processing, including quality control, cell filtration, variable gene selection, clustering, and differential expression gene (DEG) analysis of the data sets of C+F and C+F+E spheroids.

*Preprocessing of scRNA-seq data:* In the scRNA-seq analysis, a total of 4,167 cells were counted. Clustering was carried out on the cells after cell filtration to eliminate the low-quality cells. For cell filtration, thresholds were set at a minimum UMI count of 500, a minimum gene count of 300, a maximum mitochondrial ratio of 0.2, and a minimum complexity of 0.8. 335 cells from C+F and 494 cells from C+F+E spheroids passed quality control and were involved in clustering and analysis (**Figure S15**, Supporting Information).

*Clustering analysis and cell type annotation*: Highly variable genes were identified using the WinSeurat program, and principal component analysis (PCA) was performed with the top 2,000 variable genes. Clustering was conducted using the first 30 principal components (PCs), with an optimal number of 5 PCs and a resolution parameter of 3. Clusters were defined using the FindClusters function, and cells were visualized in two-dimensional space using uniform manifold approximation and projection (UMAP). Cell types for each cluster were manually annotated.

*Differential expression (DE) analysis from scRNA-seq*: DE analysis between endothelial co-cultured and non-co-cultured spheroid samples was conducted using ExSEGA (Excel-based Single Cell Expression Analysis) analysis tool provided by Ebiogen, and Cellenics. Cellenics is based on a pseudobulk limma-voom workflow to identify differentially expressed genes by grouping cells based on conditions and treating those groups as independent samples. Pseduobulk samples were created based on spheroid culture conditions (C+F and C+F+E) upon 6 different cell clusters.

*Cellular pathway analysis*: Pathway enrichment analysis was carried out using the ‘MSigDB_Hallmark_2020’ gene set library within the Enrichr software, available at https://maayanlab.cloud/Enrichr/#libraries. The considered gene set library is based upon the MSigDB database (https://www.msigbd.org) and hallmarks datasets.^[2]^ Statistical significance was assigned with p value <0.01. Analysis was done in pairwise comparisions, therefore the positive enriched pathways for one group were negative enriched pathways for the other and vice versa. The negatively enriched pathways were implicitly considered.

*Proteome Profiler Array*: Supernatants were collected after 48 hours of culture and 24 hours of treatment with 4-Hydroxytamoxifen Ready-Made Solution (213 µg mL^-1^, SML1666, Sigma) in the ODSEI chip . Samples were centrifuged to remove particulates before the assay. The Proteome Profiler Human Angiogenesis Kit (ARY007, R&D Systems, MN, USA) was used to study the expression of secreted proteins in prepared supernatant, following the protocol provided by the manufacturer. Chemiluminescent signals from the spots on the array membrane were detected using a gel imaging system (C600, Azure Biosystems, CA, USA), and signal intensities were quantified and analyzed using ImageJ software.

*Neutralization:* Neutralizing antibodies were added to the mixed culture medium for spheroids, and the spheroids were cultured on the chip for 72 hours. IL-8 (0, 1, 5, 10, 20 ng mL^-1^, MAB208-100, R&D Systems), TIMP-1 (0, 1, 5, 10, 20 ng mL^-1^, MA5-13688, Thermo Fisher), and Serpin E1 (0, 1, 5, 10, 20 ng mL^-1^, MAB1786-100, R&D Systems) neutralizing antibodies were used to target cytokines secreted by cells, with a mouse IgG antibody (0, 1, 5, 10, 20 ng mL^-1^, MAB002, R&D Systems) serving as a negative control.

*Tight junction measurement:* Tight junction imaging of endothelial cells were measured with 1:400 diluted ZO-1 monoclonal antibody (MA3-39100-A488, Thermo Fisher) in fixed samples. Cell nuclei were counterstained with 1 μM Hoechst 33342 (B2261, Merck) for 30 min and washed twice with PBS. The fluorescence images were aquired using a confocal microscopy (TCS SP8, Leica Microsystems) and processed and analyzed with ImageJ software.

*Permeability*: Permeability was assessed in co-cultures of spheroids with endothelial cells and endothelial cell-only cultures using the ODSEI platform. 70 kDa dextran conjugated with Texas Red dye (D1830, ThermoFisher) was used at the final concentration of 0.125 mg mL^-1^. The fluorescence of the dextran was imaged under 10X confocal microscopy (LSM880, Carl Zeiss). The intensity images were quantified with FIJI software, permeability from each condition was calculated with the initial slope approach to compare the effect of the endothelial layer in spheroid drug exposure.^[3]^

*Statistical Analysis:* All experiments were performed independently at least three times. The number of replicates for each experiment is specified in the figure legends. Two-tailed unpaired Student’s t-tests were used to compare the means of two groups, while one- or two-way ANOVA was used for comparisons among three or more groups, followed by Tukey’s HSD test. All statistical analyses were conducted using GraphPad Prism 9. Data are represented as mean ± standard deviation (SD). Nonsignificant (N.S.), *, **, and *** denote p-values of >0.05, <0.05, <0.01, and <0.001, respectively. A p-value of <0.05 was considered statistically significant.


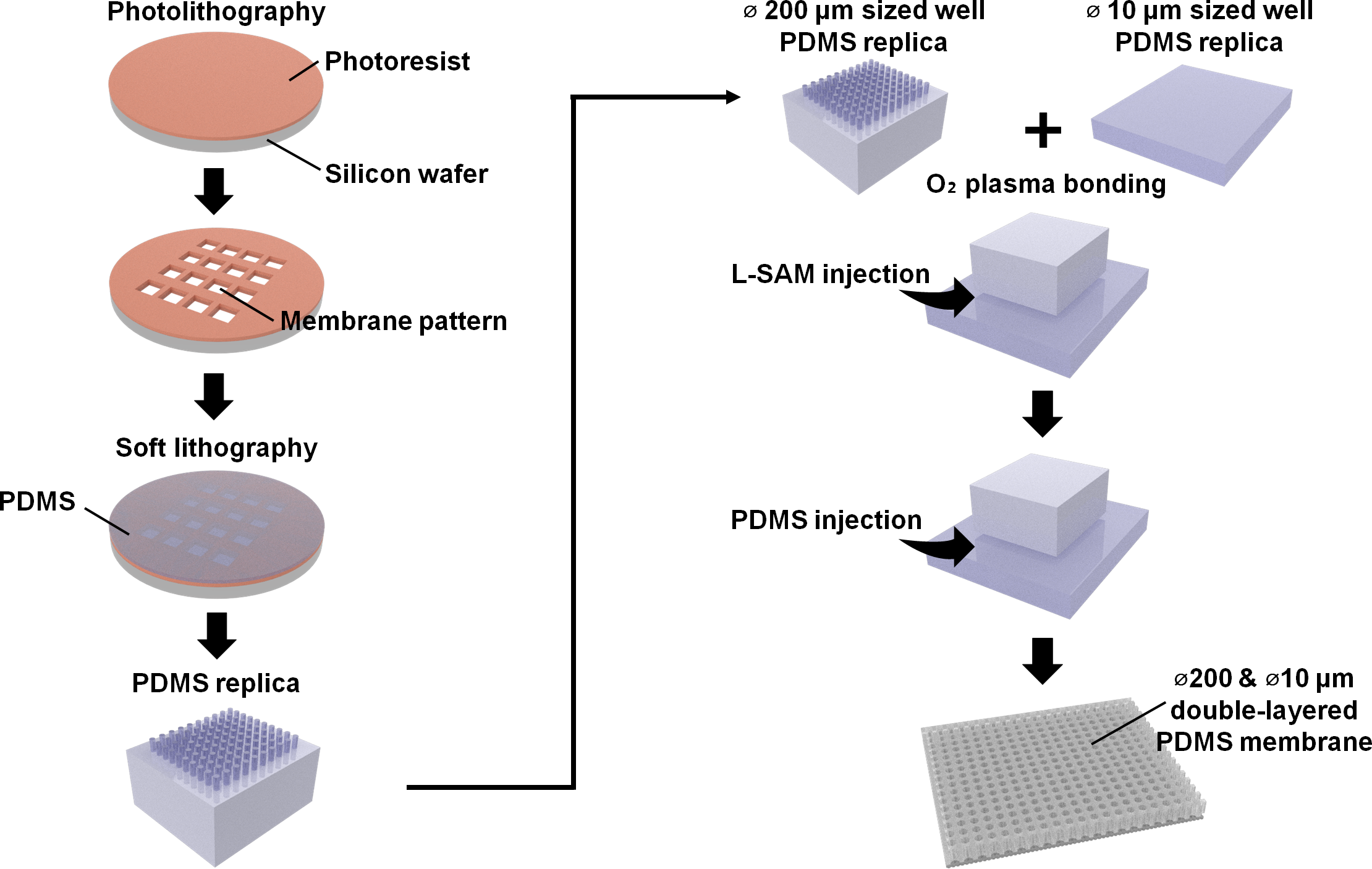


**Figure S1. The fabrication process of the double-layered PDMS membrane using photolithography and soft lithography techniques.** The fabrication of the double-layered PDMS membrane begins with photolithography, where a design is developed on a silicon wafer, creating a mold for PDMS casting. This process produces a positive relief pattern on a PDMS block. The two-layered membrane is then fabricated through soft lithography by bonding two PDMS blocks, each with a design featuring 200 µm-sized wells and 10 µm-sized wells, using oxygen plasma treatment. To facilitate the detachment of the PDMS membrane, L-SAM is applied and treated overnight. Liquid PDMS is then injected into the gap between the two bonded PDMS blocks, filling the entire interface through capillary force. The membrane is formed after curing the liquid state PDMS for 4 hours at 65℃.


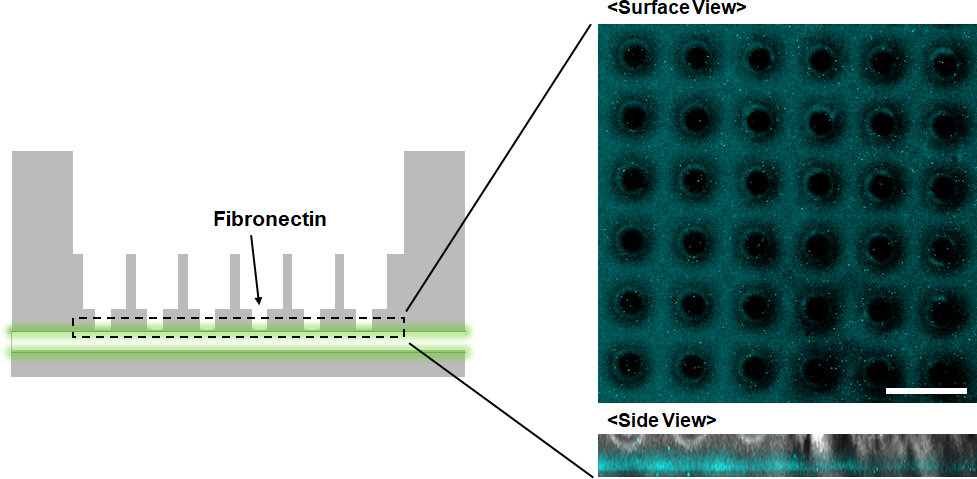


**Figure S2. Fluorescence image of the fibronectin-coated PDMS membrane bottom surface in the channel of the ODSEI chip.** Fibronectin labeled with Alexa Fluor 488 (represented in blue) was used for coating and imaging. Images were taken with 20X confocal microscope, and the side view is presented through z-stack imaging. The scale bar represents 20 µm.


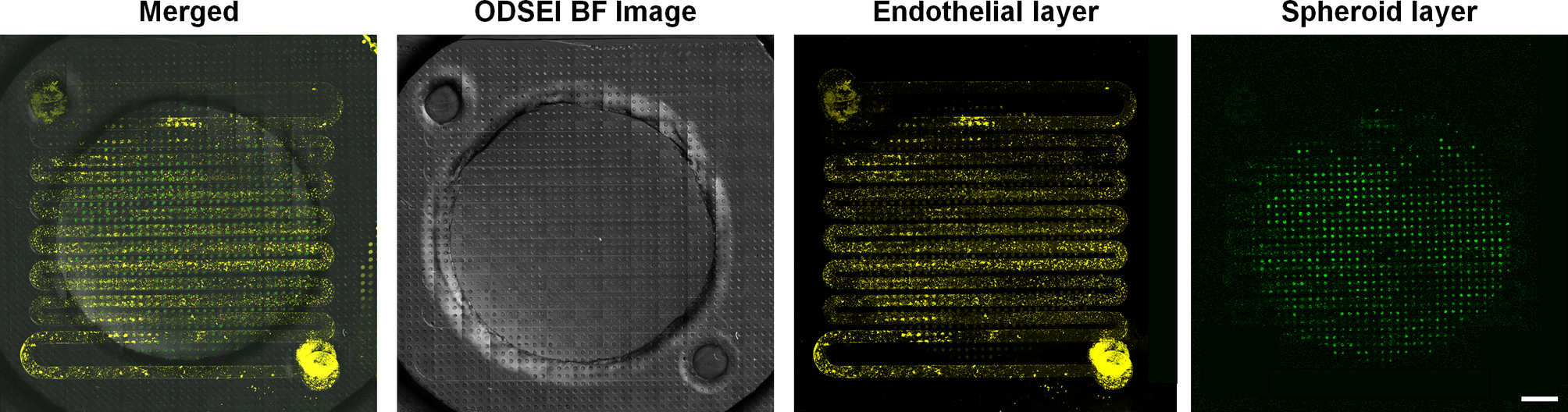


**Figure S3. Image of the entire ODSEI chip showing spheroids and endothelial cells.** Individual spheroids (green, CMFDA) formed on single spheroid wells are located on CMTMR-stained HUVECs (yellow). The whole chip was imaged using confocal microscopy with a 10X magnification lens through tile scanning. The scale bar represents 2 mm


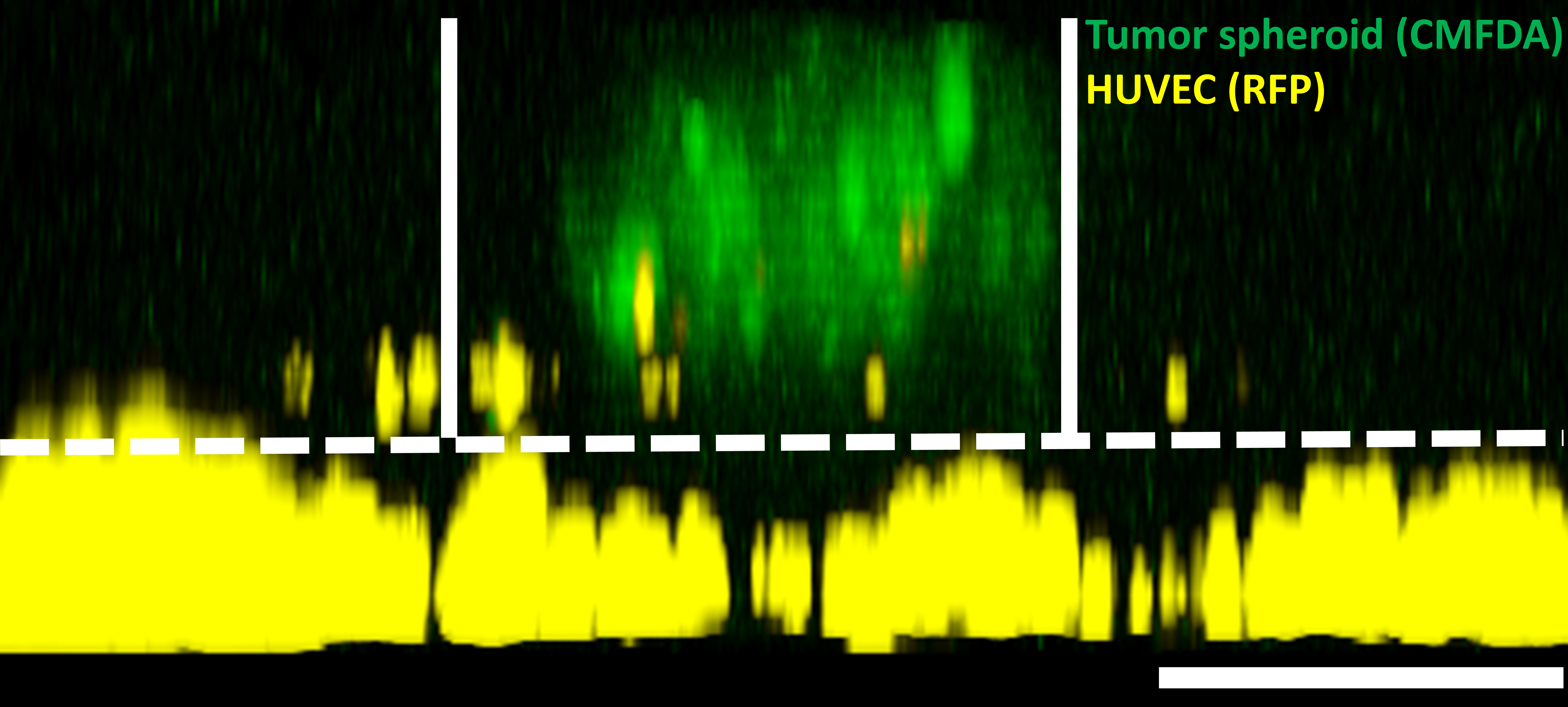


**Figure S4. Confocal microscopy images showing the interface between CMFDA-labeled tumor spheroids and RFP-expressing HUVEC layers on the ODSEI device after 7 days of culture.** The images were captured from side views through z-stack projection. The scale bar represents 100 μm.


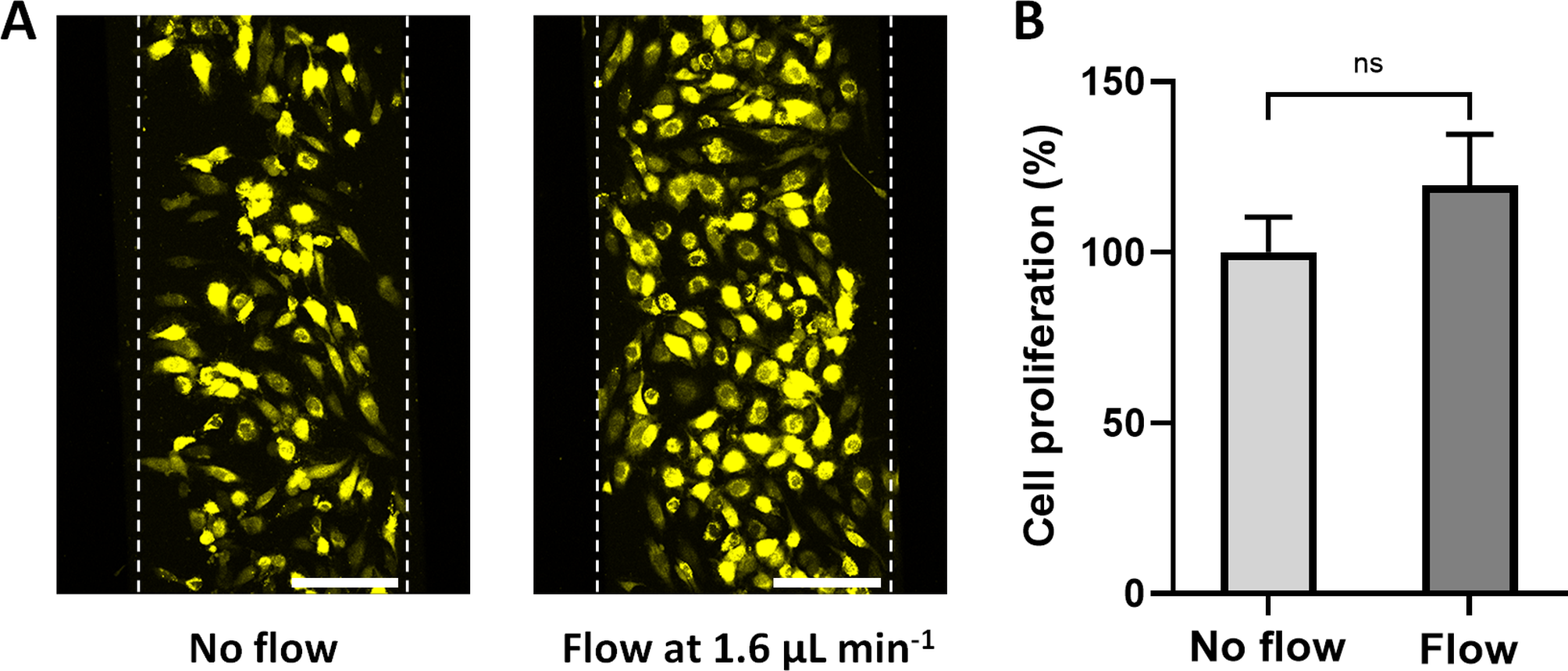


**Figure S5. HUVEC proliferation under no-flow and flow conditions in the ODSEI chip. (A)** Confocal images of RFP expressing endothelial cells (HUVECs) cultured under no-flow and flow conditions (1.6 µL min^-1^) on the ODSEI chip. The scale bar represents 200 µm. **(B)** MTT assay results comparing cell proliferation between no-flow and flow conditions. The data indicate that the presence of flow does not negatively impact endothelial cell proliferation, demonstrating that HUVEC proliferation is maintained under flow conditions. A two-tailed unpaired Student’s t-test was used for comparison; ns, p > 0.05.


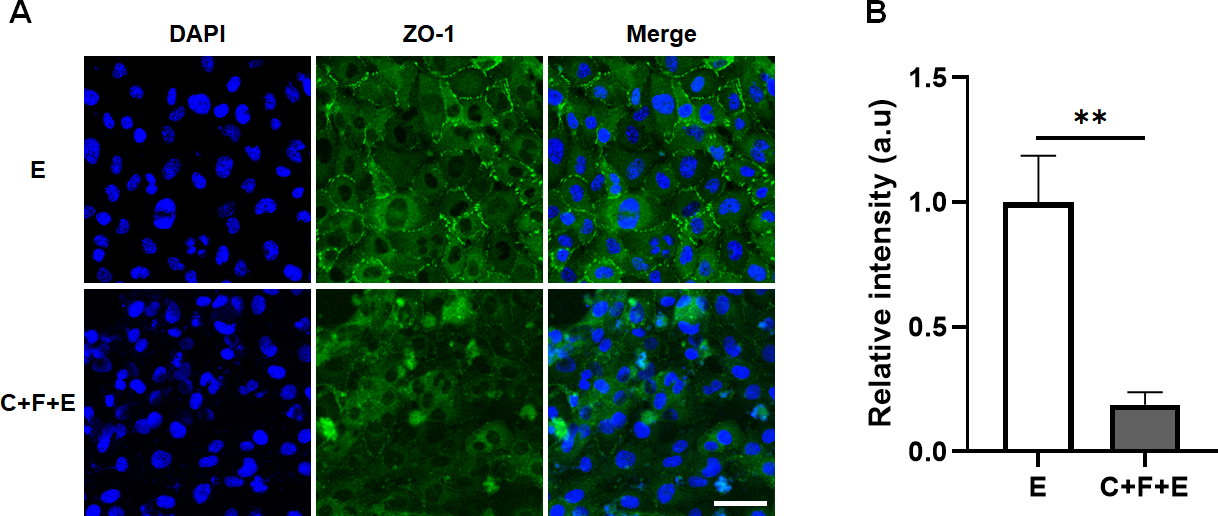


**Figure S6. ZO-1 expression in the vasculature of the ODSEI chip. (A)** Representative images and **(B)** quantification of ZO-1 (tight junction marker) and DAPI (4′,6-diamidino-2-phenylindole, nucleus marker) in endothelium under monoculture (E) and co-culture with cancer and fibroblast spheroids (C+F+E). ZO-1 is represented by green fluorescence, and DAPI by blue fluorescence in the images. The scale bar represents 50 μm. A two-tailed unpaired Student's t-test was used for comparison; **p < 0.01.


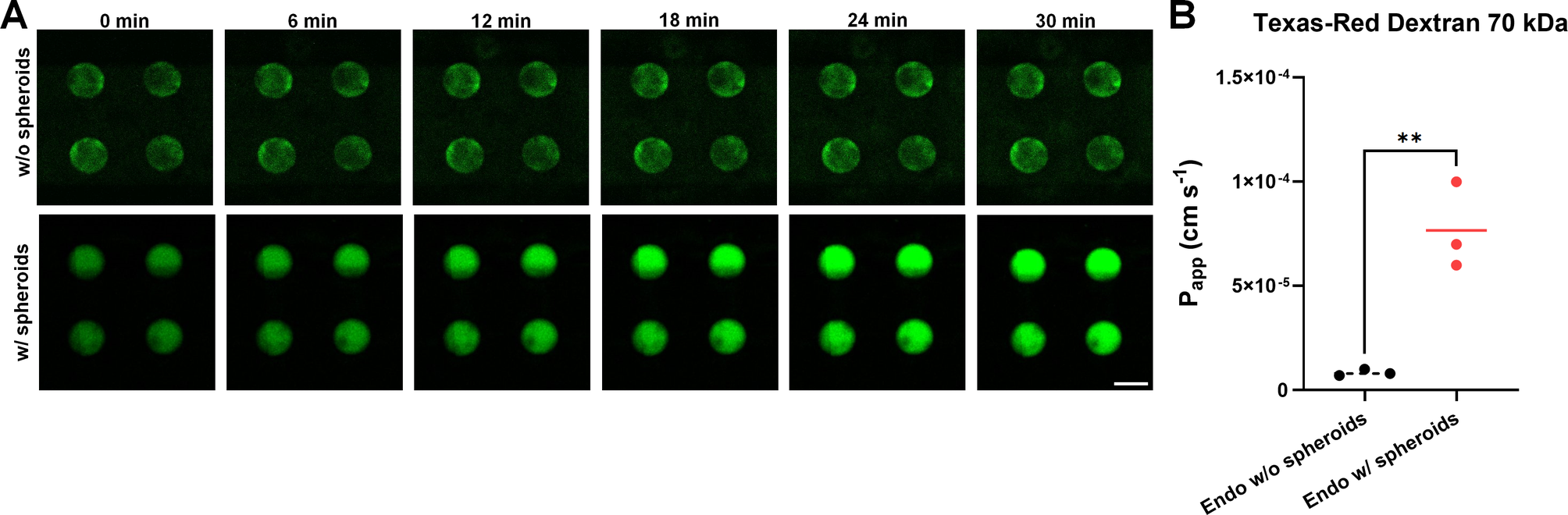


**Figure S7. Permeability test with Texas Red-conjugated dextran (70 kDa).** (A) Representative images and (B) quantification of dextran in the spheroid culturing wells under endothelial-only (w/o spheroids) conditions and co-culture of endothelial and spheroids (w/ spheroids) conditions. Dextran is represented in green fluorescence. The scale bar represents 200 μm. A two-tailed unpaired Student’s t-test was used for comparison; ****p < 0.0001.


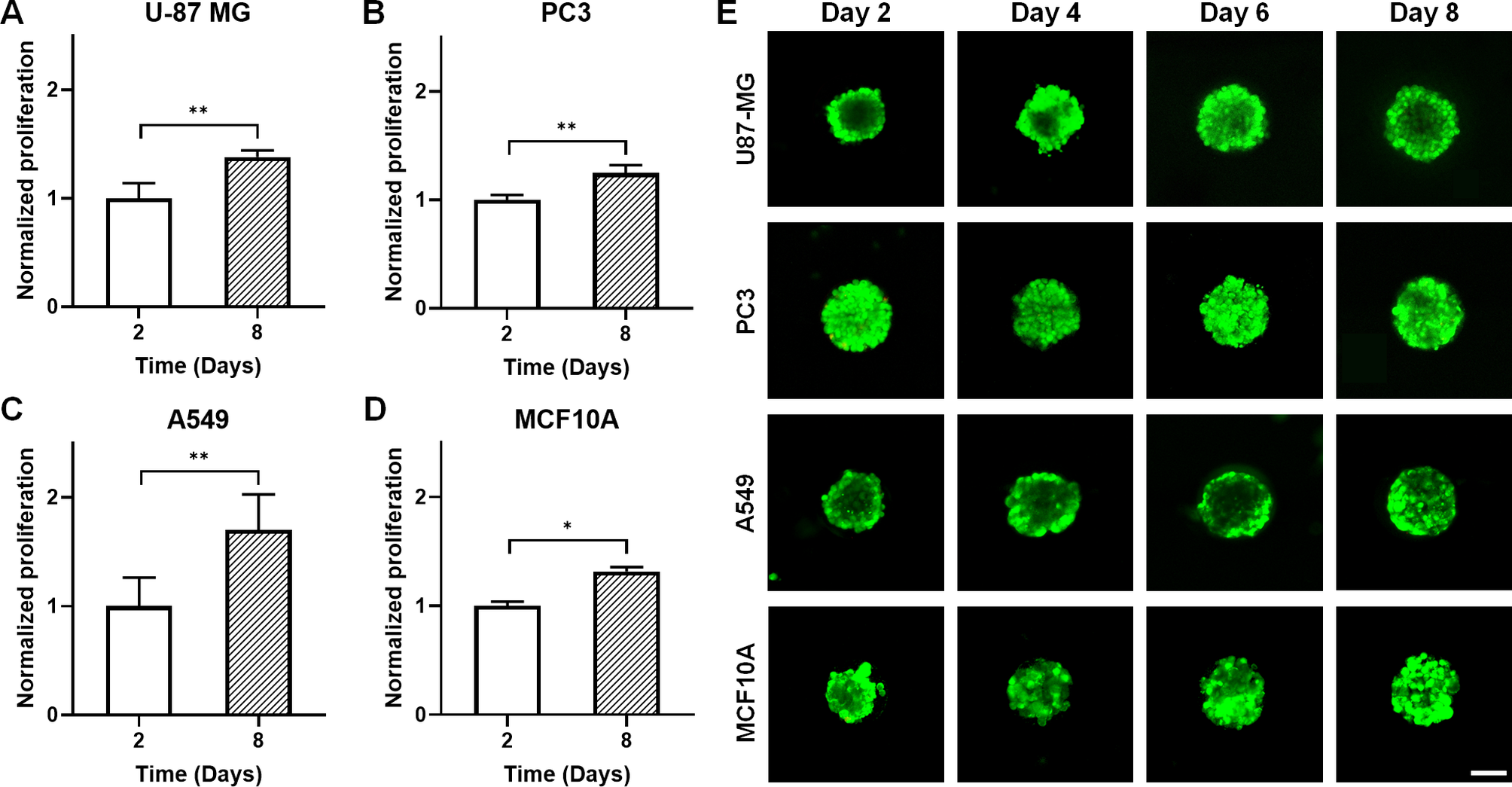


**Figure S8. Formation of spheroids from various cell lines in the ODSEI chip.** (A-D) Spheroids formed from U87-MG, PC3, A549, and MCF10A, with proliferation measurements on Days 2 and 8. (E) Images of spheroids on Days 2, 4, 6, and 8. Live/dead assays were performed using Calcein-AM (green fluorescence) for live cells and EthD-1 (red fluorescence) for dead cells. A two-tailed unpaired Student’s t-test was used for statistical comparison (p < 0.05; p < 0.01). The scale bar represents 100 μm.


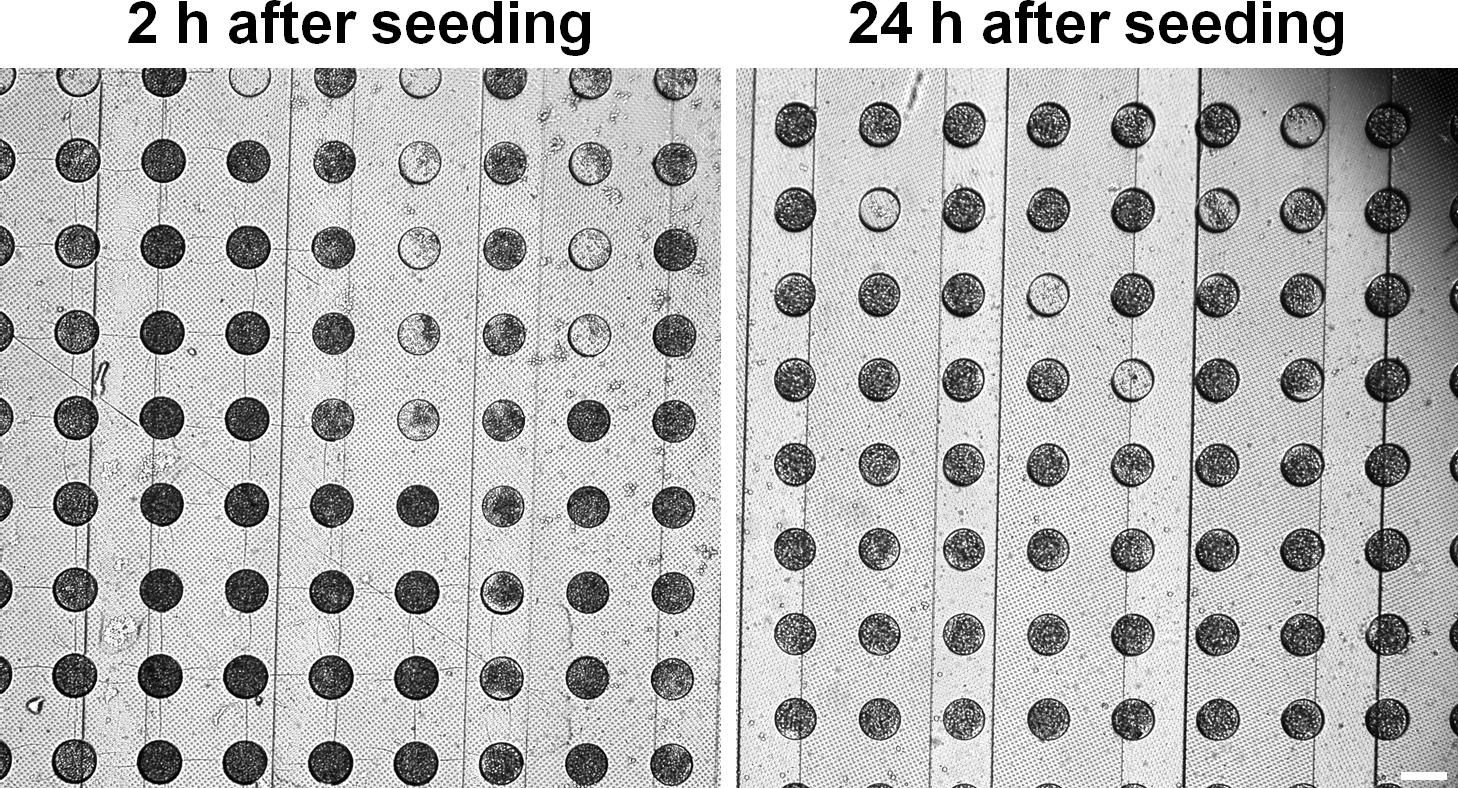


**Figure S9. Representative images of spheroid formation ratio on the ODSEI chip.** Images of spheroids formed at 2-hour and 24-hour time points on the membrane of the ODSEI chip were captured using 4X bright-field microscopy. The scale bar represents 200 μm.


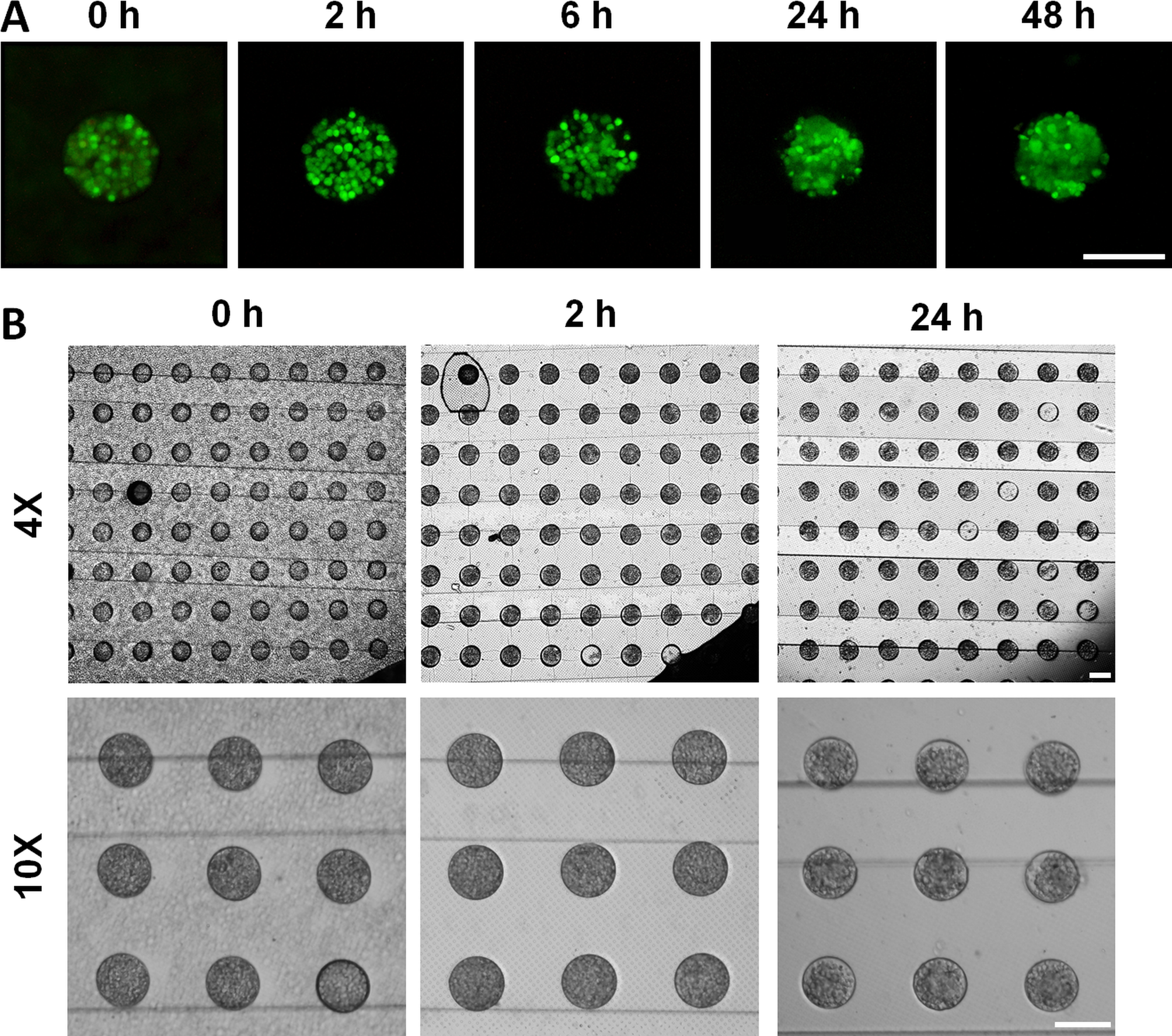


**Figure S10. Time-lapse images of self-spheroid formation in the ODSEI chip. (A)** Images of spheroid formation in the ODSEI chip were captured with green fluorescence at 0, 2, 6, 24, and 48-hour time points. **(B)** Spheroid formation on the device was visualized using 4X and 10X bright-field microscopy. All scale bars represent 200 μm.


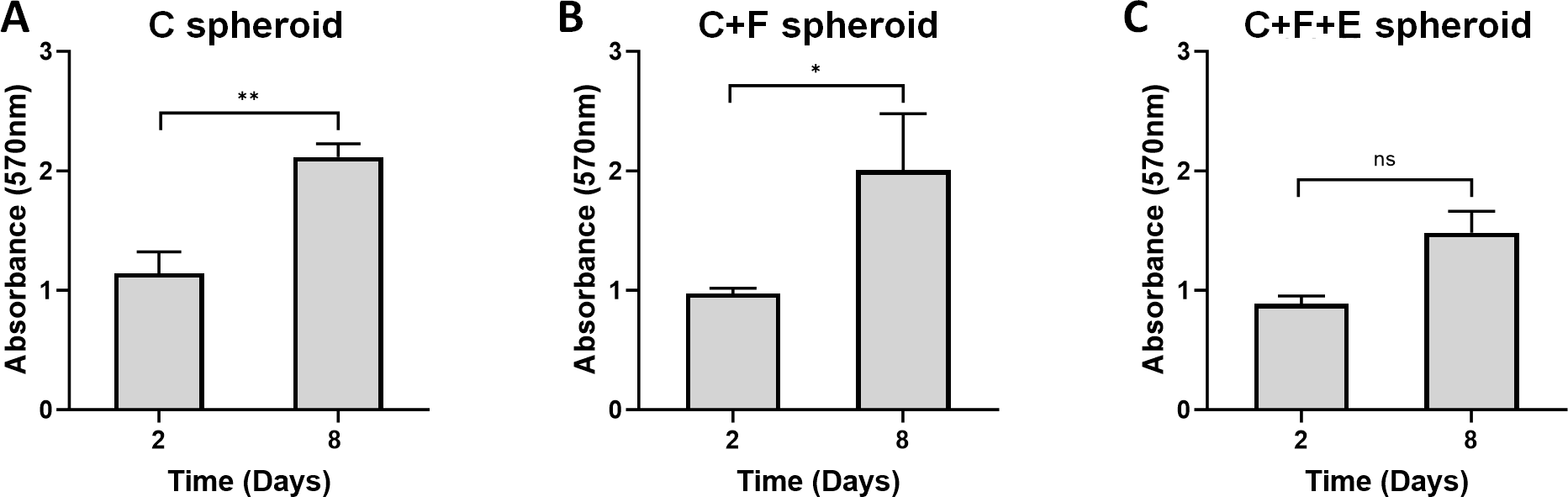


**Figure S11. Proliferation measurement of spheroids cultured under different conditions. (A)** Proliferation of cancer spheroids (C) was measured on days 2 and 8 using the MTT assay. **(B)** Proliferation of cancer and fibroblast spheroids (C+F) was measured on days 2 and 8 using the MTT assay. **(C)** Proliferation of cancer and fibroblast spheroids co-cultured with endothelial cells (C+F+E) was measured on days 2 and 8 using the MTT assay. Two-tailed unpaired Student’s t-tests was used for comparison; ns., p>0.05; *, p < 0.05; **, p < 0.01.


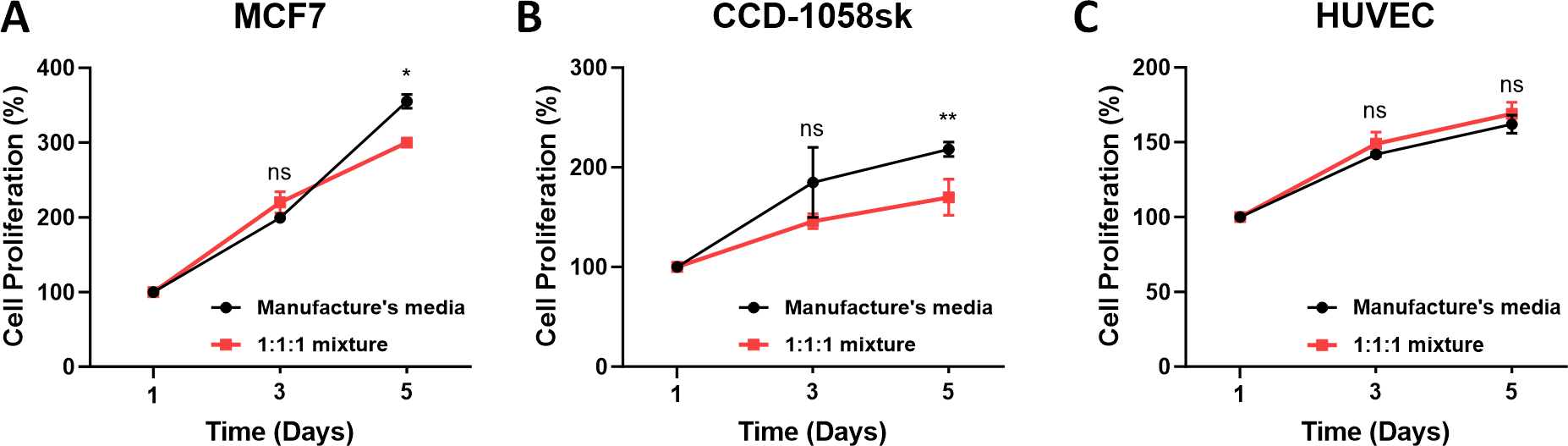


**Figure S12. Cell proliferation in mixed cell culture media.** MTT assay results show the proliferation of **(A)** MCF7 breast cancer cells, **(B)** CCD-1058sk fibroblast cells, and **(C)** HUVEC endothelial cells. Proliferation was compared between the manufacturer-recommended media for each cell line and a 1:1:1 mixture of these media. The recommended media are Roswell Park Memorial Institute (RPMI)-1640 medium for MCF7 cells, minimum essential medium (MEM) for CCD-1058sk cells, and complete human endothelial cell medium for HUVECs. The results indicate that cell proliferation was similar in both the manufacturer’s media and the 1:1:1 mixed media. Two-tailed unpaired Student’s t-tests was used for comparison; ns., p>0.05; *, p < 0.05; **, p < 0.01.


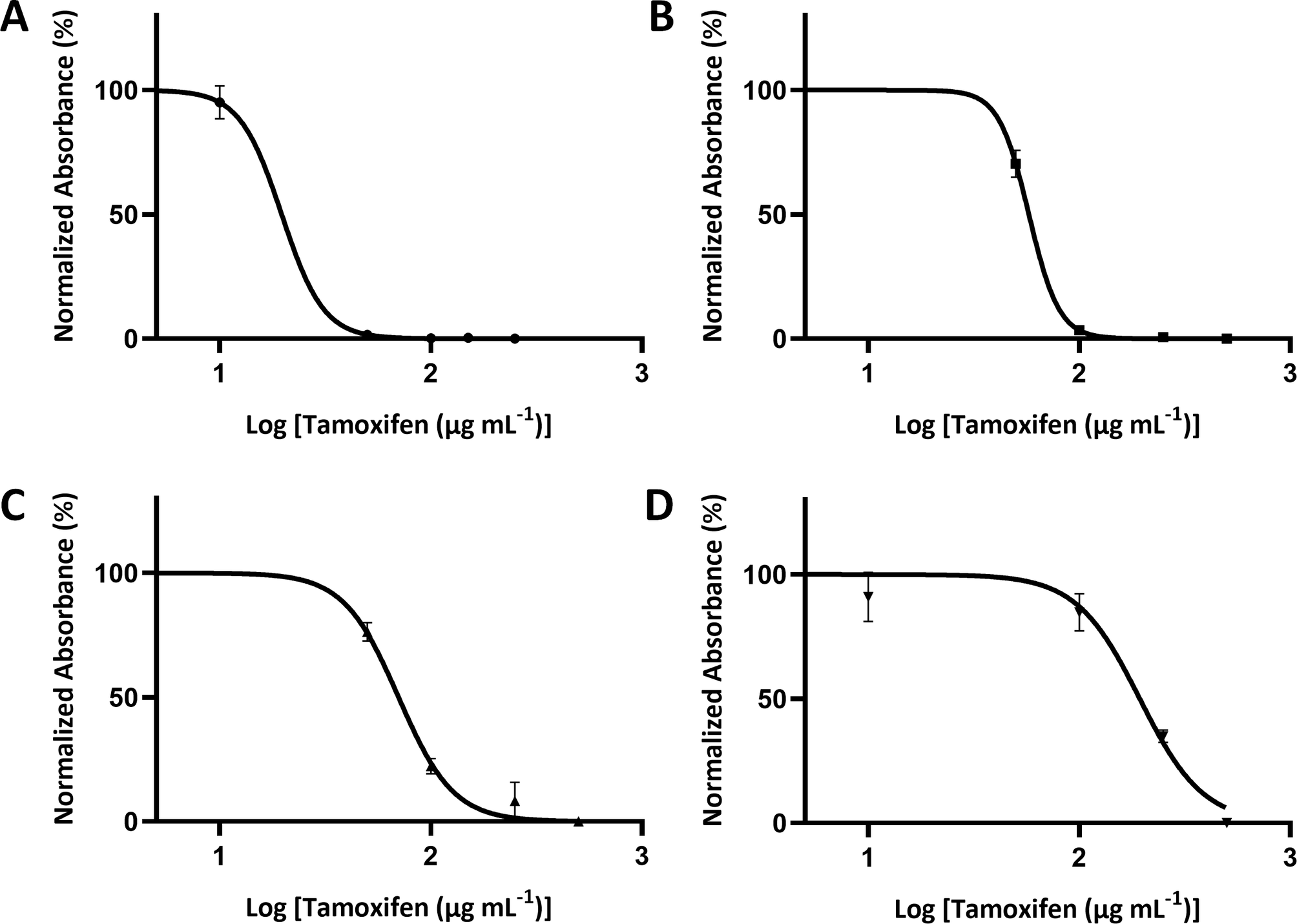


**Figure S13. Determination of IC_50_ values for each culture condition using the MTT assay. (A)** Monolayer cultured MCF7, **(B)** MCF7 cancer spheroid (C), **(C)** cancer and fibroblast spheroids (C+F), **(D)** cancer and fibroblast spheroids co-cultured with endothelial cells (C+F+E). The IC_50_ values for each condition are **(A)** 19.63 μg mL^-1^, **(B)** 57.65 μg mL^-1^, **(C)** 70.24 μg mL^-1^, and **(D)** 213.3 μg mL^-1^, respectively.


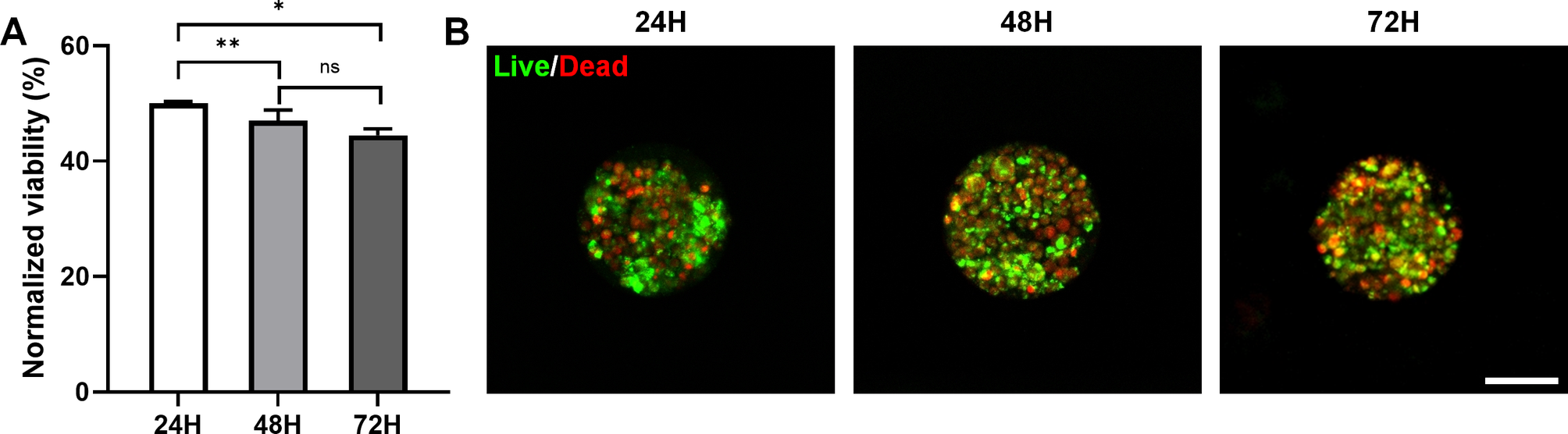


**Figure S14. Extended tamoxifen toxicity measurement of tumor spheroids. (A)** Tumor spheroid viability was measured over 72 hours at 24-hour intervals using the MTT assay. **(B)** Live/dead assay of spheroids at different time points, using Calcein-AM (green fluorescence for live cells) and EthD-1 (red fluorescence for dead cells). One-way ANOVA was used for comparison; ns., p>0.05; *, p<0.05; **, p<0.01.


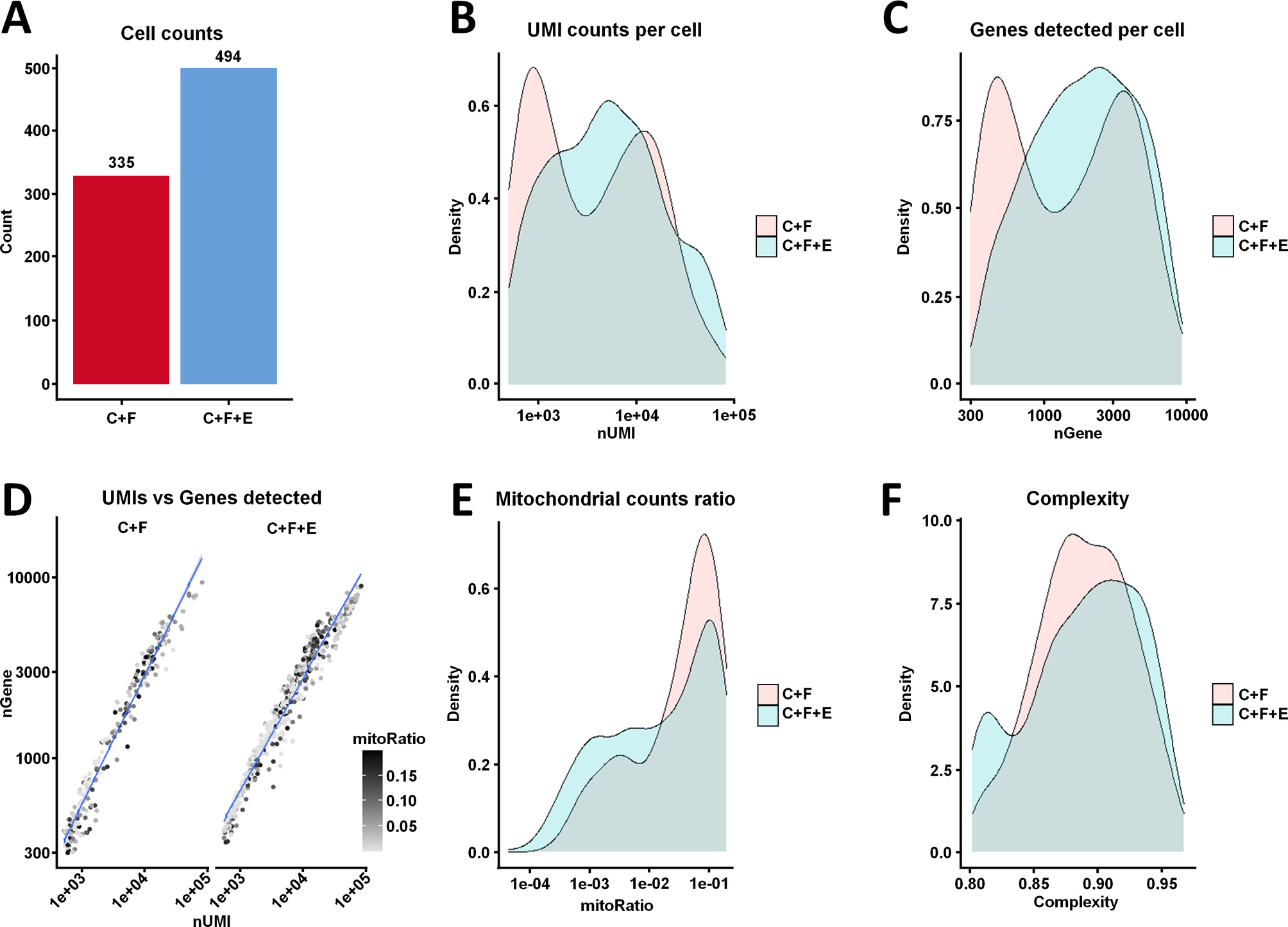


**Figure S15. Quality control of sc-RNA sequencing data.** Graphs showing various quality control metrics for single-cell RNA sequencing data: **(A)** filtrated measures of cell counts, **(B)** UMI (Unique Molecular Identifier) counts per cell, **(C)** number of genes detected per cell, **(D)** scatter plot comparing UMI counts and the number of genes detected per cell, **(E)** mitochondrial gene count ratio, indicating the proportion of mitochondrial transcripts, and **(F)** complexity, measured as the ratio of unique genes to total transcripts, reflecting the diversity of gene expression within each cell.


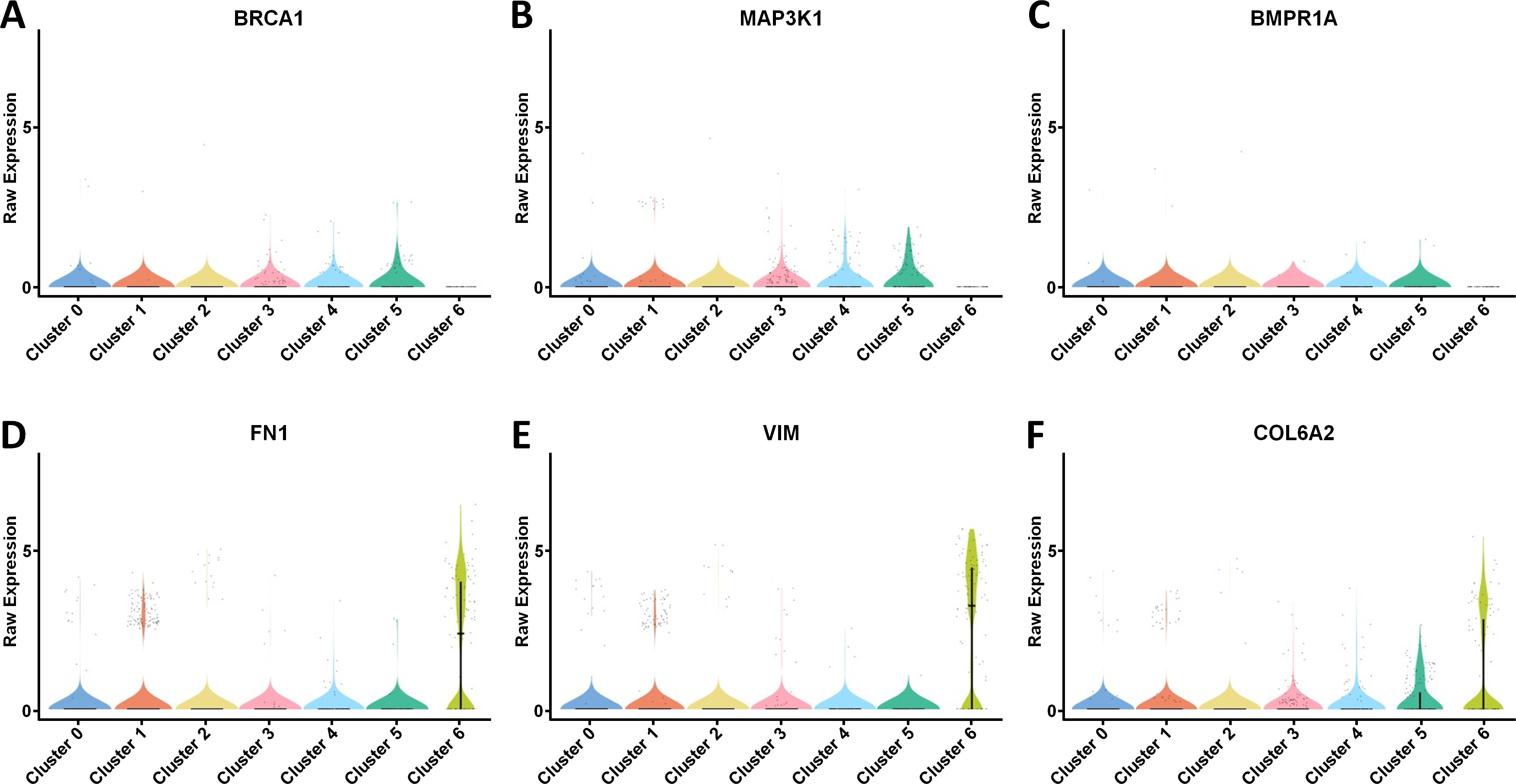


**Figure S16. Violin plots of marker gene expression in breast cancer and fibroblast cells after quality control.** Violin plots displaying the expression levels of recognized marker genes: **(A-C)** Breast cancer cell markers BRCA1, MAP3K1, and BMPR1A observed in clusters 0-5. **(D-F)** Fibroblast cell markers VIM, FN1, and LGALS were observed in cluster 6.


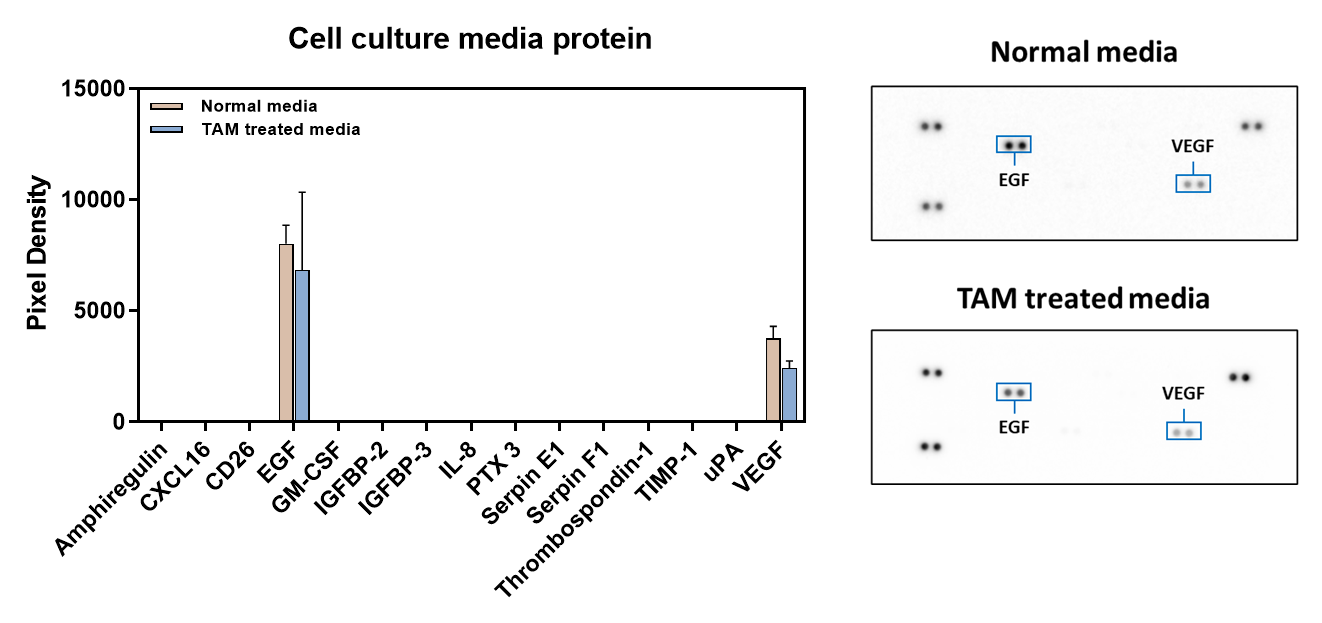


**Figure S17.** **Cytokine array from mixed cell culture media.** Protein array analysis was conducted to identify proteins present in the 1:1:1 mixed cell culture medium used for co-cultured spheroids. Both EGF and VEGF were detected in all experimental conditions. The levels of EGF and VEGF were subtracted from the final protein expression data to ensure an accurate interpretation of the results. This figure provides supporting data for **Figure 5C**.


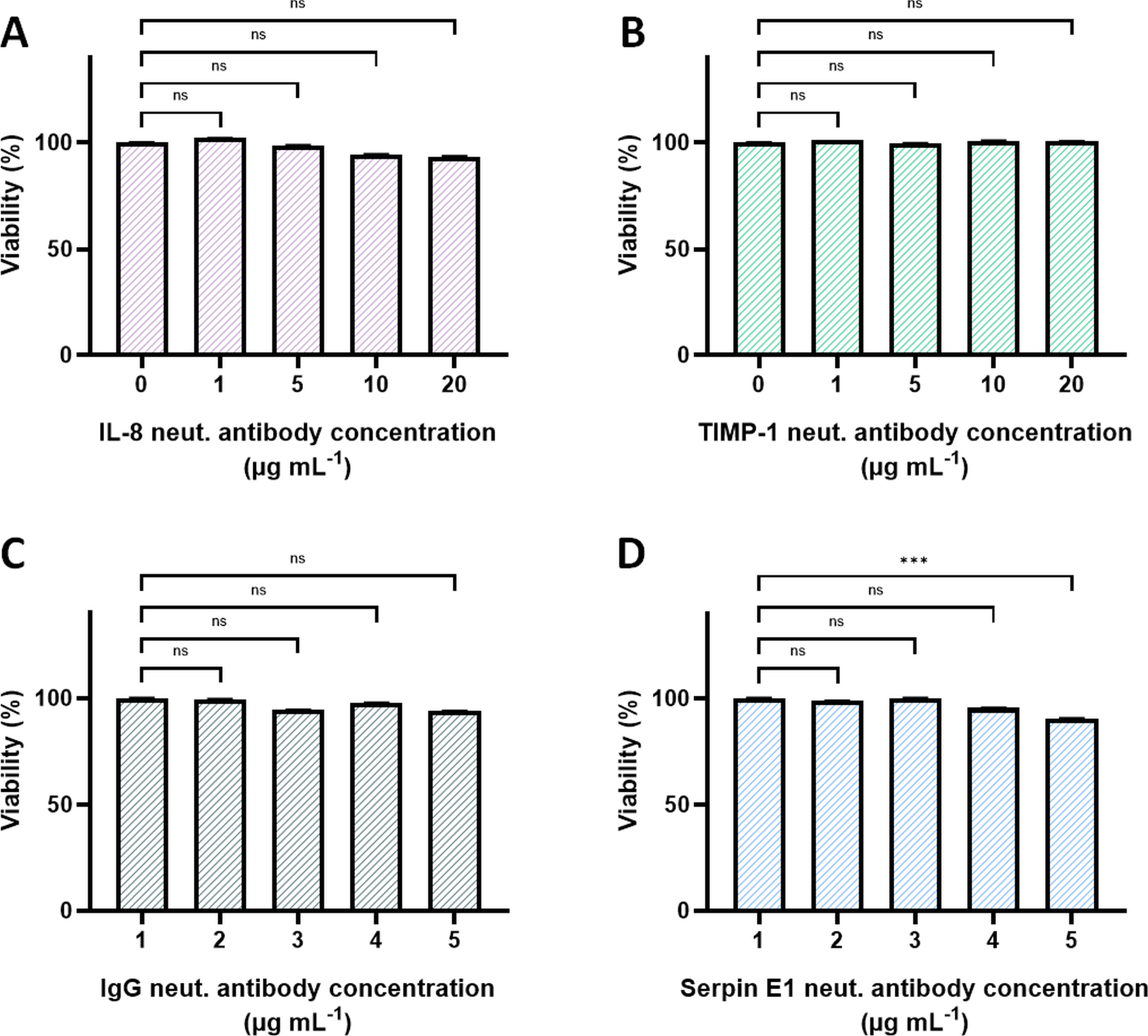


**Figure S18. Viability of spheroids treated with neutralizing antibodies (Supporting data for Figure 5).** The viability of spheroids treated only with neutralizing antibodies was compared to the control group (0 µg mL⁻¹ concentration). Statistical analysis was performed using one-way ANOVA; ns, p > 0.05; ***, p < 0.001.

**Figure S19. Neutralization of Serpin E1 expression in the ODSEI chip.** Comparison of tamoxifen cytotoxicity with the neutralization of Serpin E1 using a neutralizing antibody, followed by treatment with 213 μg mL^-1^ of tamoxifen. One-way ANOVA was used for comparison; ns., p>0.05; **p < 0.01, and ***p < 0.001.


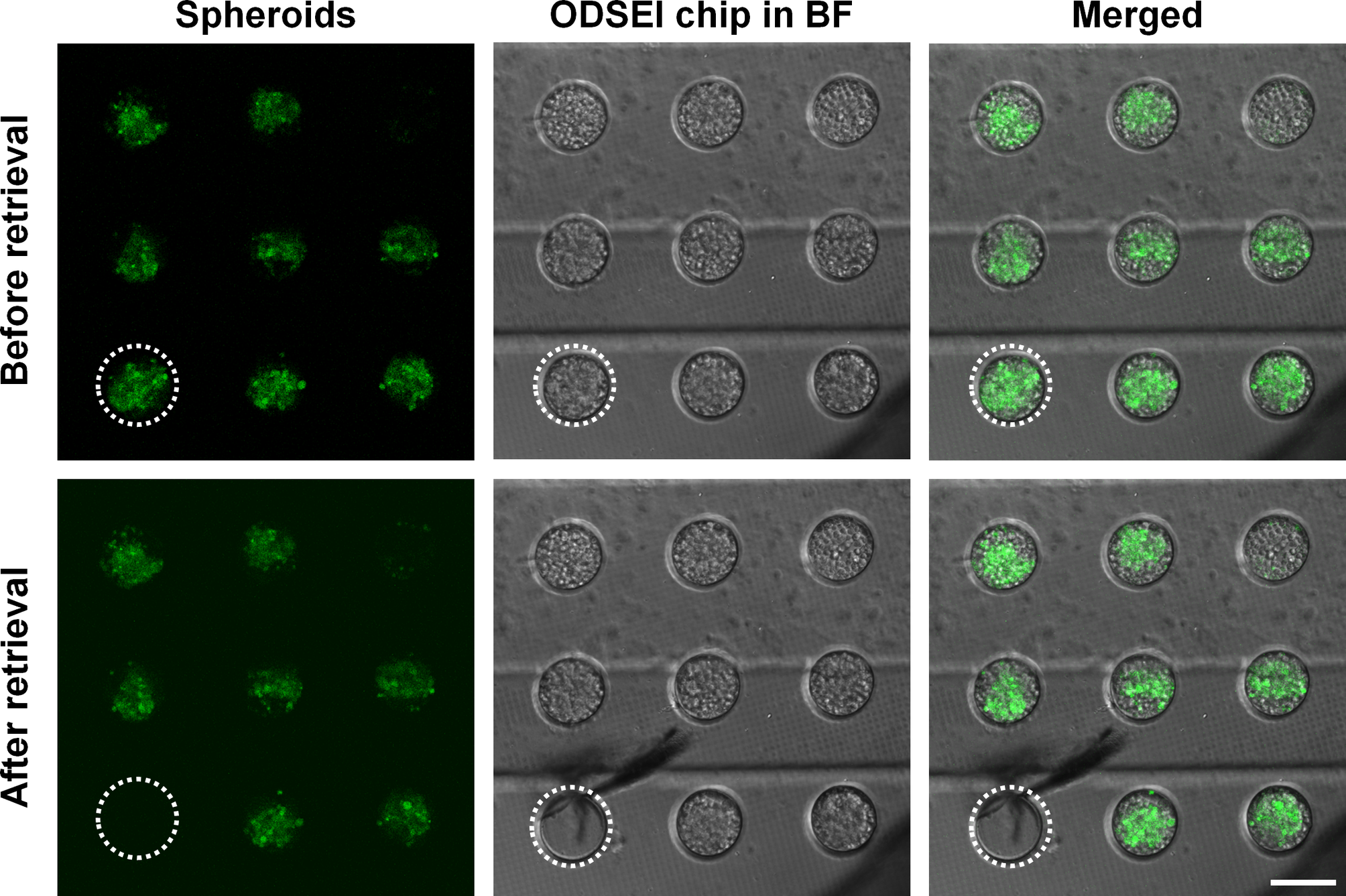


**Figure S20. Specific retrieval of spheroid from the ODSEI chip.** Using a cell picker system, specific spheroids can be located and retrieved from the device. Spheroids were labeled with CellTracker Green CMFDA and imaged using 10X confocal microscopy. The scale bar represents 200 μm. (BF: Bright Field)

**Table S1**. Comparison with other vascularized tumor-on-a-chips.^[4]^

| Fabrication type | Ref | Cell types | Formation  of cancer | Vessel  (E, C, L) | Interaction  (P, C) | Throughput  (L, M, H) | Addressable positioning | Selective spheroid retrieval | Biological phenomena |
| --- | --- | --- | --- | --- | --- | --- | --- | --- | --- |
| MP_P | Our system | Cancer (MCF7, PC3, A549, U87), HUVEC, CCD 1058sk | Heterocellular spheroid (SA) | E | P, C | H (~1,000) | O^#^ | O | Drug screening |
| MP_M | [4a] | Cancer (A549, MDA-MB-231, M624, BEL-7402), Primary BMEC, Astrocyte | Suspended cells | E | P, C | L (NA) | NA | X | Organotrophic metastasis |
|  | [4b] | Cancer (MCF7), HUVEC | Monocellular spheroid (PP) | E | P, C | M (NA) | X^#^ | X | Tumor extravasation |
| MP_P | [4c] | Cancer (MDA-MB-231), HDMEC | Suspended cells | E | P, C | NA | NA | NA | Cancer adhesion |
|  | [4d] | Cancer (H1975), airway epithelial cell, lung microvascular endothelial cell | Suspended cells | E | P, C | NA | NA | NA | Organotypic cancer |
|  | [4c] | Cancer (HepG2, MDA-MB-231), HUVEC or EAhy926 | Epithelium | E | P, C | NA | NA | NA | Tumor progression, Drug screening |
|  | [4f] | Cancer (A549), HUVEC, W138, THP-1, HA-1800, Fob1.19, L-02 | Epithelium | E | P, C | NA | NA | NA | Organotrophic metastasis |
|  | [4g] | Cancer (GSC), HBMEC or Eahy926 | Monocellular spheroid (SA) | E | C | M (NA) | X^#^ | X | Drug screening |
|  | [4h] | Cancer (SKOV3), HUVEC | Monocellular spheroid (PP) | E | C | M (NA) | X^#^ | X | Nanoparticle delivery |
| MP_S^c^ | [4i] | Cancer (HT1080),  HMVEC | Suspended cells | E | P, C | NA | NA | NA | Tumor intravasation |
|  | [4j] | Cancer (MDA-MB-231)  HMVEC | Suspended cells | E | P, C | NA | NA | NA | Tumor extravasation |
|  | [4k] | Cancer (MDA-MB-231, A-375 MA2, 4T1), HUVEC, hLF | Suspended cells | E | P, C | NA | NA | NA | Tumor extravasation |
|  | [4l] | Cancer (MDA-MB-231), HUVEC,  BM-MSC | Suspended cells | E | P, C | NA | NA | NA | Organotrophic metastasis |
|  | [4m] | Cancer (MDA-MB-231), HUVEC, MSC | Suspended cells | C | P, C | NA | NA | NA | Organotrophic metastasis |
|  | [4n] | Cancer (MDA-MB-231), HUVEC,  BM-MSC, OD-MSC | Suspended cells | C | P, C | NA | NA | NA | Organotrophic metastasis |
|  | [4o] | Cancer (A549), HUVEC, human monocyte or macrophage | Monocellular spheroid (PP) | E | C | M (NA) | X^#^ | X | Immune infiltration |
|  | [4p] | Cancer (HeLa), HUVEC, hLF | Heterocellular spheroid (PP) | C | P, C | L (1) | NA | X | Tumor progression |
|  | [4q] | Cancer (SW620, HCT116, U87MG, HepG2, A549, PDC), HUVEC, hLF | Heterocellular spheroid (SA) | C | P, C | L (8) | O^#^ | O | Drug screening |
|  | [4r] | Cancer (HepG2), HUVEC, IMR90 | Heterocellular spheroid (PP) | C | P, C | M (NA) | X^#^ | X | Drug screening |
|  | [4s] | Cancer (MDA-MB-231), HUVEC, hLF, MSC | Heterocellular spheroid (PP) | L | P, C | M (NA) | X^#^ | X | Tumor-induced angiogenesis |
| ST | [4t] | Cancer (Mouse and human primary cancer), HUVEC | Heterocellular spheroid (PP) | L | P, C | M (NA) | X^#^ | X | Tumor intravasation |
|  | [4u] | Cancer (MDA-MB-231, MCF7),  iPSC-EC | Suspended cells | L | P, C | NA | NA | NA | Tumor extravasation |
|  | [4v] | Cancer (MCF7), HUVEC, NK-92 | Gel-embedded cells | L | P, C | NA | NA | X | Immune exhaustion |
| 3P | [4w] | Cancer (A549), HUVEC, hDF | Gel-embedded cells | L | P, C | NA | NA | NA | Tumor progression, Drug screening |
|  | [4x] | Cancer (MDA-MB-231), HUVEC | Gel-embedded cells or Monocellular spheroid (PP) | L | P, C | NA | NA | NA | Angiogenesis, Tumor intravasation |
| MP_P, MP_S | [4y] | Cancer (U87MG), HDMEC, hLF | Monocellular spheroid (PP) | C | P, C | L (1) | NA | O | Angiogenesis |
| ST,  MP_S | [4z] | Cancer (A549), HUVEC, hLF | Heterocellular spheroid (PP) | L, C | P, C | M (NA) | X^#^ | O | Drug screening |

MP_M: Microfluidic platform (Monolayer), MP_P: Microfluidic platform (Porous membrane), MP_S: Microfluidic platform (Self-organization), 3P: 3D printing, ST: Sacrificial template, SA: Self-assembled, PP: Pre-prepared, E: Endothelium, C: Capillary, L: Lumen, P: Physical interaction, C: Chemical interaction, NA: Not available or applicable to add information, L (#): Low-throughput with # spheroids per chip, M (NA): Mid-throughput with 10 < throughput <100 spheroids per chip, H (1,000): High-throughput with ~1,000 spheroids per chip, O^#^ : Addressable and aligned spheroid positioning, X^#^: Randomized spheroid positioning, O*: Selective spheroid retrieval is possible, X*: Selective spheroid retrieval is impossible.

**Reference**

[1] H. E. Karakas, J. Kim, J. Park, J. M. Oh, Y. Choi, D. Gozuacik, Y.-K. Cho, *Scientific Reports* **2017**, *7* (1), 2050, https://doi.org/10.1038/s41598-017-02172-7.

[2] A. Liberzon, C. Birger, H. Thorvaldsdottir, M. Ghandi, J. P. Mesirov, P. Tamayo, *Cell Syst* **2015**, *1* (6), 417, https://doi.org/10.1016/j.cels.2015.12.004.

[3] C. Soragni, T. Vergroesen, N. Hettema, G. Rabussier, H. L. Lanz, S. J. Trietsch, L. J. de Windt, C. P. Ng, *STAR Protocols* **2023**, *4* (1), 102051, https://doi.org/https://doi.org/10.1016/j.xpro.2023.102051.

[4] a) H. Xu, Z. Li, Y. Yu, S. Sizdahkhani, W. S. Ho, F. Yin, L. Wang, G. Zhu, M. Zhang, L. Jiang, Z. Zhuang, J. Qin, *Sci Rep* **2016**, *6*, 36670, https://doi.org/10.1038/srep36670; b) Y. Zhang, F. Jiang, Y. C. Zhao, A. N. Cho, G. Fang, C. D. Cox, H. Zreiqat, Z. F. Lu, H. Lu, L. A. Ju, *Biomed Mater* **2023**, *18* (5), https://doi.org/10.1088/1748-605X/ace7a4; c) J. W. Song, S. P. Cavnar, A. C. Walker, K. E. Luker, M. Gupta, Y. C. Tung, G. D. Luker, S. Takayama, *PLoS One* **2009**, *4* (6), e5756, https://doi.org/10.1371/journal.pone.0005756; d) B. A. Hassell, G. Goyal, E. Lee, A. Sontheimer-Phelps, O. Levy, C. S. Chen, D. E. Ingber, *Cell Rep* **2017**, *21* (2), 508, https://doi.org/10.1016/j.celrep.2017.09.043; e) B. Jing, Y. Luo, B. Lin, J. Li, Z. A. Wang, Y. Du, *RSC Advances* **2019**, *9* (30), 17137, https://doi.org/10.1039/C9RA02069A; f) Z. Xu, E. Li, Z. Guo, R. Yu, H. Hao, Y. Xu, Z. Sun, X. Li, J. Lyu, Q. Wang, *ACS Appl Mater Interfaces* **2016**, *8* (39), 25840, https://doi.org/10.1021/acsami.6b08746; g) C. Lin, L. Lin, S. Mao, L. Yang, L. Yi, X. Lin, J. Wang, Z. X. Lin, J. M. Lin, *Anal Chem* **2018**, *90* (17), 10326, https://doi.org/10.1021/acs.analchem.8b02133; h) H.-F. Wang, R. Ran, Y. Liu, Y. Hui, B. Zeng, D. Chen, D. A. Weitz, C.-X. Zhao, *ACS Nano* **2018**, *12* (11), 11600, https://doi.org/10.1021/acsnano.8b06846; i) I. K. Zervantonakis, S. K. Hughes-Alford, J. L. Charest, J. S. Condeelis, F. B. Gertler, R. D. Kamm, *Proc Natl Acad Sci U S A* **2012**, *109* (34), 13515, https://doi.org/10.1073/pnas.1210182109; j) J. S. Jeon, I. K. Zervantonakis, S. Chung, R. D. Kamm, J. L. Charest, *PLoS One* **2013**, *8* (2), e56910, https://doi.org/10.1371/journal.pone.0056910; k) M. B. Chen, J. M. Lamar, R. Li, R. O. Hynes, R. D. Kamm, *Cancer Res* **2016**, *76* (9), 2513, https://doi.org/10.1158/0008-5472.CAN-15-1325; l) S. Bersini, J. S. Jeon, G. Dubini, C. Arrigoni, S. Chung, J. L. Charest, M. Moretti, R. D. Kamm, *Biomaterials* **2014**, *35* (8), 2454, https://doi.org/10.1016/j.biomaterials.2013.11.050; m) A. Marturano-Kruik, M. M. Nava, K. Yeager, A. Chramiec, L. Hao, S. Robinson, E. Guo, M. T. Raimondi, G. Vunjak-Novakovic, *Proc Natl Acad Sci U S A* **2018**, *115* (6), 1256, https://doi.org/10.1073/pnas.1714282115; n) J. S. Jeon, S. Bersini, M. Gilardi, G. Dubini, J. L. Charest, M. Moretti, R. D. Kamm, *Proc Natl Acad Sci U S A* **2015**, *112* (1), 214, https://doi.org/10.1073/pnas.1417115112; o) J. Bai, G. Adriani, T. M. Dang, T. Y. Tu, H. X. Penny, S. C. Wong, R. D. Kamm, J. P. Thiery, *Oncotarget* **2015**, *6* (28), 25295, https://doi.org/10.18632/oncotarget.4716; p) C. Li, S. Li, K. Du, P. Li, B. Qiu, W. Ding, *ACS Appl Mater Interfaces* **2021**, *13* (17), 19768, https://doi.org/10.1021/acsami.1c03740; q) Y. Kim, J. Ko, N. Shin, S. Park, S. R. Lee, S. Kim, J. Song, S. Lee, K. S. Kang, J. Lee, N. L. Jeon, *Biotechnol Bioeng* **2022**, *119* (12), 3678, https://doi.org/10.1002/bit.28221; r) J. Park, S. Kim, J. Hong, J. S. Jeon, *Lab Chip* **2022**, *22* (22), 4335, https://doi.org/10.1039/d2lc00597b; s) T. J. Kwak, E. Lee, *Sci Rep* **2020**, *10* (1), 20142, https://doi.org/10.1038/s41598-020-77180-1; t) V. L. Silvestri, E. Henriet, R. M. Linville, A. D. Wong, P. C. Searson, A. J. Ewald, *Cancer Res* **2020**, *80* (19), 4288, https://doi.org/10.1158/0008-5472.CAN-19-1564; u) M. Humayun, J. M. Ayuso, R. A. Brenneke, M. Virumbrales-Munoz, K. Lugo-Cintron, S. Kerr, S. M. Ponik, D. J. Beebe, *Biomaterials* **2021**, *270*, 120640, https://doi.org/10.1016/j.biomaterials.2020.120640; v) J. M. Ayuso, S. Rehman, M. Virumbrales-Munoz, P. H. McMinn, P. Geiger, C. Fitzgerald, T. Heaster, M. C. Skala, D. J. Beebe, *Science Advances* **2021**, *7* (8), eabc2331, https://doi.org/doi:10.1126/sciadv.abc2331; w) F. Meng, C. M. Meyer, D. Joung, D. A. Vallera, M. C. McAlpine, A. Panoskaltsis-Mortari, *Adv Mater* **2019**, *31* (10), e1806899, https://doi.org/10.1002/adma.201806899; x) J. Nie, Q. Gao, C. Xie, S. Lv, J. Qiu, Y. Liu, M. Guo, R. Guo, J. Fu, Y. He, *Materials Horizons* **2020**, *7* (1), 82, https://doi.org/10.1039/c9mh01283d; y) S. Oh, H. Ryu, D. Tahk, J. Ko, Y. Chung, H. K. Lee, T. R. Lee, N. L. Jeon, *Lab on a Chip* **2017**, *17* (20), 3405, https://doi.org/10.1039/C7LC00646B; z) J. Paek, S. E. Park, Q. Lu, K.-T. Park, M. Cho, J. M. Oh, K. W. Kwon, Y.-s. Yi, J. W. Song, H. I. Edelstein, J. Ishibashi, W. Yang, J. W. Myerson, R. Y. Kiseleva, P. Aprelev, E. D. Hood, D. Stambolian, P. Seale, V. R. Muzykantov, D. Huh, *ACS Nano* **2019**, *13* (7), 7627, https://doi.org/10.1021/acsnano.9b00686.
